# Global Landscape of Total Organic Carbon, Nitrogen and Phosphorus in Lake Water

**DOI:** 10.1038/srep15043

**Published:** 2015-10-19

**Authors:** Ming Chen, Guangming Zeng, Jiachao Zhang, Piao Xu, Anwei Chen, Lunhui Lu

**Affiliations:** 1 College of Environmental Science and Engineering, Hunan University, Changsha 410082, China; 2 Key Laboratory of Environmental Biology and Pollution Control (Hunan University), Ministry of Education, Changsha 410082, China; 3 College of Resources and Environment, Hunan Agricultural University, Changsha 410128, China

## Abstract

Human activities continue to increase the amount of carbon (C), nitrogen (N) and phosphorus (P) in lakes, which may cause serious environmental and human health problems. Global landscape of total organic C (TOC), N and P in lake water is still poorly known. Using a global data set that covers ~8300 lakes from 68 countries/regions spanning six continents, we estimate that global mean concentrations and storage in lake water are 5.578 mg L^−1^ and 984.0 Tg for TOC, 0.526 mg L^−1^ and 92.8 Tg for TN, and 0.014 mg L^−1^ and 2.5 Tg for TP. These lake elements are significantly interrelated and in uneven distribution, being associated with morphological characteristics and climate conditions. We proposed that global C, N and P cycles should be considered as a whole in biogeochemical studies and policy-making related to environmental protection.

Fossil fuel combustion, economic development, the use of pesticides and fertilizers, the increased request for carbon (C), nitrogen (N) and phosphorus (P) in human activities and land-use change are transforming global C, N and P cycles, and bring serious environmental threats, including global warming, eutrophication, biodiversity decline, water quality deterioration and air pollution^1^,^2^,^3^,^4^,^5^,^6^. Lakes have a critical role in the global cycling of C, N and P^1^,^7^, although they only account for a small fraction of the Earth^8^. TOC, TN and TP once enter the lakes, and may remain in water. TOC, TN and TP are also likely to be transferred from lake water to bottom as sediments by biological, physical and chemical processes^1^,^9^. These elements retained in sediments may be released again into lake water as an important internal C, N and P sources^10^. Processes in lakes such as exchange, transport, sedimentation, fixation, nitrification, denitrification and interaction may change water quality and biogeochemical cycles^1^,^2^.

Here, we presented comprehensive estimates of mean concentration, storage and interrelationship of TOC, TN and TP for the world’s lakes based on national lake survey, journal publications and unpublished documents. We advanced our estimates by including 1 to multiple sample sites and 1 to several sample dates for each lake of ~8300 lakes from 68 countries/regions (Table S1) across six continents representing a wide range of climatic conditions. We not only analyzed mean TOC, TN and TP concentrations in lake water, but also reported their ratios. We further subdivided lakes into deep lakes (average depth (AD) >10 m) and shallow lakes (AD ≤ 10 m). Using the global data set, we searched for general relationships between these lake elements and climatic variables as well as chlorophyll *a* (Chl *a*), focusing on the influence of lakes’ morphological characteristics on these elements.

## Materials and Methods

### Data collection

Data was collected from national lake survey, journal publications and unpublished documents. These documents were obtained by searching for ‘carbon, nitrogen, phosphorus or Chl *a*’ + ‘country’ + ‘lake’ in Google Scholar or Google. The data included by Sobek *et al.*^11^ and Gu *et al.*^12^ were selectively collected for the present study. Physical parameters of lakes including area, maximum depth (MD), average depth (AD) and geographical coordinates were considered in our global data set. In particular, these parameters were not always simultaneously available for each lake. In some cases, we only got the area parameter of a lake, although we collected large numbers of related literature. Climate data (mean annual temperature, MAT; mean annual precipitation, MAP; relative humidity, RH; sunshine, SUN) were retrieved from the University of East Anglia Climatic Research Unit (http://www.cru.uea.ac.uk/data). Some of these climate data have been used for the analyses of climate controls on lake DOC concentrations^11^ and soil microbial biomass C and N^13^. In cases where the geographical coordinate of a lake was not given, it can be obtained on the basis of information on the sites^13^. Geographical coordinate of each lake served as an index to extract climate data from the selected database.

### TOC, TN, TP or Chl *a* and other parameters

TOC, TN, TP or Chl *a* concentrations given in a range (minimum-maximum) were excluded from the analysis because mean concentrations of TOC, TN, TP or Chl *a* could not be estimated only relying on the minimum and maximum of concentrations. Lakes treated by lime, nutrient addition or other anthropogenic activities were not considered due to that anthropogenic interference would change physico-chemical characteristic of lakes. C species included IC and OC. TOC often serves as an indirect measure of organic matter. It is also often used as an indicator of organic pollution.

When DOC concentration (C_DOC_) was known whereas TOC concentrations (C_TOC_) was unknown, C_DOC_ was converted to C_TOC_ by dividing by 0.9^11^:
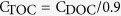


C, N, P and Chl *a* concentrations in the same lake varied between different sample times and between different sample depths or sites, respectively. The assessment of these elements was unreliable if not taking into account the temporal and spatial heterogeneities of sampling. Other problems for the assessment, such as data format and methodological differences, were also presented. Thus, mean concentrations of TOC, TN and TP were assessed for each lake with 1 up to multiple time points and 1 to multiple sample sties. The units were standardized: mg L^−1^ for TOC, TP, TN and Chl *a*, km^2^ for lake area, and m for lake AD and MD. TP concentrations were used to assess trophic state of lakes: eutrophic (>0.03 mg L^−1^), mesotrophic (0.01–0.03 mg L^−1^) and oligotrophic (<0.01 mg L^−1^)^14^.

The present global dataset included ~8300 lakes from 68 countries/regions, spanning six continents. The latitudes of the lakes whose coordinates are available ranged from 69.52° S to 81.33° N, and longitudes from 138.23° W to 176.83° E. The data set also covered a wide climatic condition, and multiple climate zones including the south temperate zone, the north temperate zone, the tropical zone and the north frigid zone. The south temperate zone located between 23.5° S and 66.5° S, the north temperate zone between 23.5° N and 66.5° N, the tropical zone between 23.5° S and 23.5° N, and the north frigid zone between 66.5° N and 90° N. The measure of C, N and P concentrations in global lakes is important in exploring biogeochemical cycles. We calculated the average concentrations of C, N and P in lake water at a global scale. Concentrations of Chl *a* and the correlation between lake elements and Chl *a* were also estimated. We did not estimate mean TOC, TN and TP concentrations for the tropical zone and the south frigid zone, and TOC and TN concentrations for the south temperate zone due to low data availability. Global storage of TOC, TN and TP in lake water was estimated by assuming that the volume of global lake water was 176 400 km^3 ^15.

### Duplicate elimination

The issue of duplicate for the data derived from various documents might appear, and must be addressed. The method of eliminating duplicate records was as follows: first, all data records were stored in a file to bring all repeated records together; then a simple algorithm was used to eliminate all but one of the repeated records. Records with the same lake name and similar limnological characteristics would be identified as the duplicate record. Noteworthy, some lakes had the same name, but their limnological characteristics differed significantly, we considered these lakes as different lakes in such a situation.

### Statistical analyses

The concentrations of TOC, TN, TP and their ratios were log_10_-transformed to meet a normal distribution, as described by Xu *et al.*^13^ and Li *et al.*^16^. Chl *a* concentration, MD, AD and area were also log_10_-transformed due to high skewness. We transformed mean TOC, TN and TP, their ratios, Chl *a* concentration, and 95% confidence intervals into original values for reporting. Statistical models of TOC, TN and TP considered the influences of Chl *a*, morphological characteristics and climate variables. Each of these three factors was analyzed separately to reveal each factor how to correlate with global C, N and P concentrations. The interrelationship between TOC, TN and TP, together with the effect of Chl *a* on lake elements was analyzed using simple linear regression. Multiple linear regression was then applied to assess lake elements’ response to the change of morphological characteristics and climate variables, taking into account the collinearity diagnostics based on tolerance values (tolerance <1-*R*^*2*^ meaning that collinearity may exist). The best model was selected according to significance, adjusted *R*^*2*^ and Akaike information criterion (AIC).

## Results and Discussion

### Mean concentrations and storage of TOC, TN and TP in global lake water

**Figure 1:**
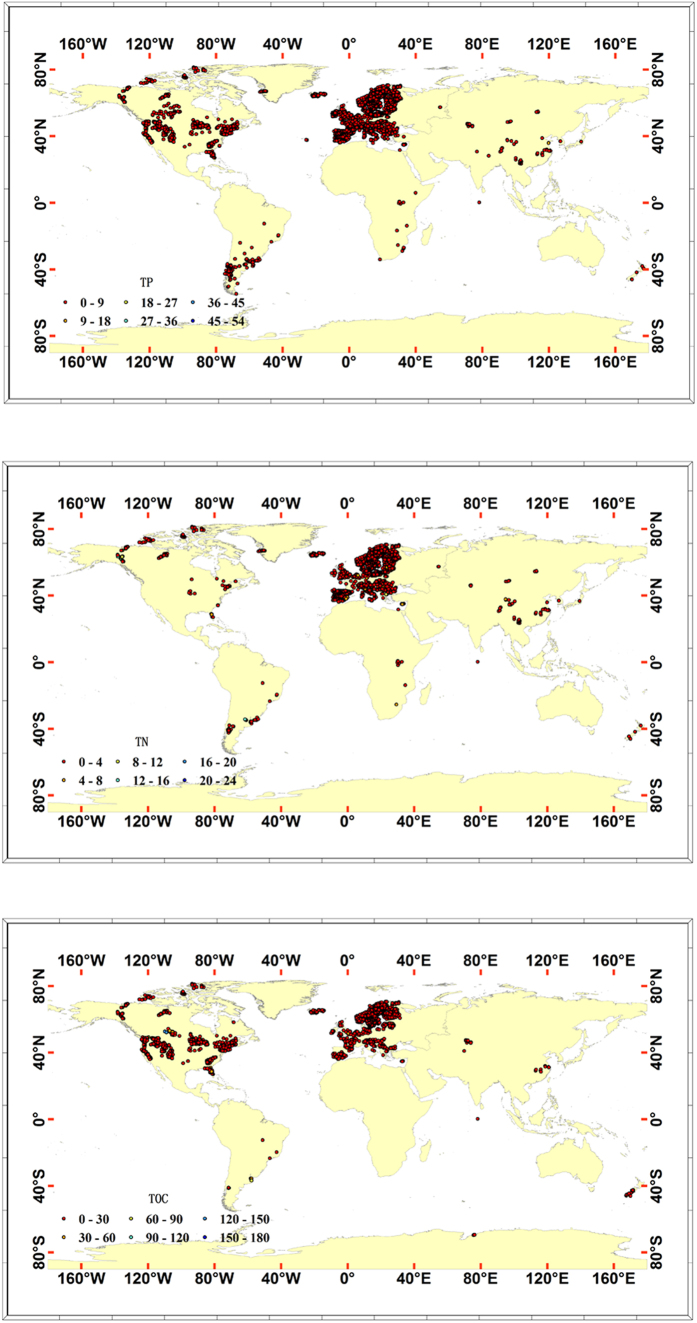
Global distribution of TP, TN and TOC concentrations in lake water. The number of lakes with available geographical coordinates is 7760 for TP, 4916 for TN and 5954 for TOC, respectively. Concentrations of TP, TN and TOC are in mg L^−1^. This figure is produced using ArcGIS 10.0.

Mean concentrations of TOC, TN and TP in global lake water were estimated to be 5.578 mg L^−1^, 0.526 mg L^−1^ and 0.014 mg L^−1^, respectively (Table 1 and Figure S1). Global storage of TOC, TN and TP in lake water was estimated to be 984.0 Tg C, 92.8 Tg N and 2.5 Tg P respectively, by assuming that the global volume of lake water was 176 400 km^3^^15^. At the level of climate zone (Figure S2), the north frigid zone had the lowest mean TOC (3.690 mg L^−1^), TN (0.322 mg L^−1^) and TP (0.00797 mg L^−1^) concentrations in lake water; the south temperate zone lake water had a mean TP concentration of 0.033 mg L^−1^; mean TOC, TN and TP concentrations in lake water of the north temperate zone were 5.809 mg L^−1^, 0.560 mg L^−1^ and 0.014 mg L^−1^, respectively. These results showed mean concentrations of TOC, TN and TP in lakes varied across climate zone. It was clear that TOC, TN and TP were distributed in unevenness in the world (Fig. 1). Among the lake types divided by AD (Figure S3), shallow lakes had higher TOC (7.291 mg L^−1^, n = 548), TN (0.893 mg L^−1^, n = 946) and TP (0.034 mg L^−1^, n = 1228) concentrations. Deep lakes had lower TOC (3.463 mg L^−1^, n = 137), TN (0.611 mg L^−1^, n = 264) and TP (0.017 mg L^−1^, n = 383) concentrations. Deep and shallow lakes had significantly different TOC (t = 9.512, *P* < 0.001) and TN (t = 3.596, *P* < 0.001) concentrations. However, this difference was not significant for TP (t = 1.618, *P* > 0.05).

### TOC:TN:TP stoichiometry in global lake water

The C:N:P stoichiometry in lakes and marine is a key feature related to biogeochemical cycling^17^. There are extensive studies focusing on the C:N:P ratio in these two ecosystems. The classical Redfield ratio of C:N:P (106:16:1)^18^ in the seston was often adopted in the ocean. Unlike the ocean, C:N:P ratio in lake particulate matter varied widely^17^. However, few studies reported the TOC:TN:TP stoichiometry in lake water on a global basis. Our study reported that mean TOC:TN, TOC:TP and TN:TP in global lake water were 21, 1467 and 74, respectively (Table 2). This result showed that lake water was generally richer in OC relative to N and P, and generally richer in N relative to P. N:P ratios of lake nutrient sources from ground water, precipitation, river water, sewage and fertilizer were found to highly vary^19^, which might partly lead to various N:P ratio in lake water. Sources of TOC are also diverse, such as internal production and the surrounding environment^2^,^20^. This might also be one of the reasons that TOC:TN or TOC:TP were in a wide range in lake water. Average TN:TP ratio was lower in eutrophic lakes than that in mesotrophic and oligotrophic lakes (35 vs. 71 vs. 129) (Table 2 and Figure S4). The dominance of TN:TP in oligotrophic lakes agreed well with a previous study^19^ which supported the idea that oligotrophic lakes received natural sources with high N:P ratio while mesotrophic and eutrophic lakes obtained nutrient sources that were complex and in low N:P ratio. This dominance was also true for TOC:TP ratios which were 2185, 1387 and 375 in oligotrophic, mesotrophic and eutrophic lakes, respectively (Table 2 and Figure S5). Although inorganic carbon (IC) was not considered in this study, our result could provide strong evidence that C:N:P stoichiometry in lake water was inconsistent with that of seston in marine and freshwater^17^. In that study, mean C:P ratio was as low as 129 but as high as 329 in lake seston, 108–171 in coastal zone, and as low as 101 but as high as 235 in oceans; in lake seston, mean N:P ratios ranged from 17 to 39, in coastal zone from 15 to 19, and in oceans from 15 to 26; mean C:N ratios in seston of lakes, coastal zone and oceans were 7.8–10.5, 7.6–9.9 and 6.5–9.3, respectively^17^.

### Relationships between TOC, TN, TP, Chl *a*, morphological characteristics and climate variables on a global basis

**Figure 2:**
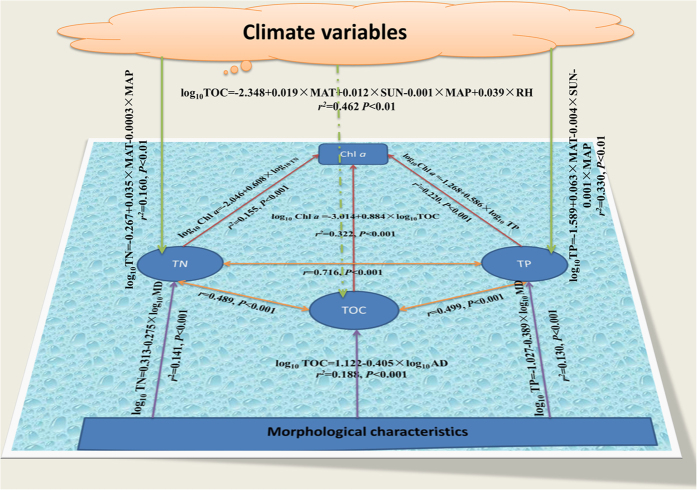
Regression equations relating Chl *a* (mg L^−1^) to TOC (mg L^−1^), TN (mg L^−1^) and TP (mg L^−1^) concentrations, and the influence of morphological characteristics and climate variables on these elements in lake water at a global scale. Chl *a*, Chlorophyll *a*; MD, maximum depth (m); AD, average depth (m); MAT, mean annual temperature (°C), MAP, mean annual precipitation (mm); SUN, sunshine (percent of maximum possible); RH, relative humidity (%).

Various factors have been found to be related to C, N and P concentrations, including Chl *a*, climate, morphological characteristics, changes in land use, climate and anthropogenic activities^11^,^21^,^22^,^23^,^24^. Based on data availability, we investigated the influence of morphological characteristics and climate variables on TOC, TN and TP concentrations in lake water, respectively. The relationships between TOC, TN, TP and Chl *a* in lake water were analyzed using linear regression. TOC, TN and TP were profoundly interrelated (Fig. 2 and Figure S6), meaning that any a change in C, N and P cycles caused by external and internal factors would result in variations in all these three cycles. We thus proposed that C, N and P cycles should be considered as a whole when concerning biogeochemical cycling. Mean concentration and storage of Chl *a* was 0.0066 mg L^−1^ and 1.2 Tg, respectively (Table 1). Globally, the association of TOC, TN and TP in lakes with Chl *a* was comparatively small (*R*^*2*^ = 0.322 for TOC; *R*^*2*^ = 0.155 for TN; *R*^*2*^ = 0.220 for TP), respectively. However, it was correlated significantly with each of these elements (*P* < 0.001) (Figure S7). The effects of morphological characteristics on TOC, TN and TP were assessed. Lake area did not correlate significantly with TOC and TP respectively (*P* > 0.05), but significantly related to TN (*P* < 0.001) (Figures S8–S10). There were weak, but significant relationships between maximum/average depth and lake elements (*P* < 0.001). Using depth as predictor, multiple regression models explained 18.8% of the variance in TOC concentration (F = 93.772, *P* < 0.001), 14.1% in TN concentrations (F = 150.149, *P* < 0.001) and 13% in TP concentrations (F = 175.794, *P* < 0.001), respectively. Other variables that may affect these elements were not fitted in our models. For example, proportion of wetlands was related to OC concentrations^25^, but corresponding data was not sufficiently available.

Climatic controls on TOC, TN and TP in lake water were determined using multiple linear regression (Fig. 2). For TOC, the best model included mean annual temperature (MAT), mean annual precipitation (MAP), sunshine (SUN) and relative humidity (RH) as predictors. All of these variables were significantly correlated with TOC concentrations (*P* < 0.05) (Figure S11), together accounting for 46.2% of the variance (F = 88.17, *P* < 0.01). Climatic factors were also important for the TN concentrations in lake water (Figure S12), especially MAT and MAP which collectively explained 16.0% of the variance (F = 46.73, *P* < 0.01). TP concentrations in lake water was significantly correlated with MAT (*P* < 0.01) and MAP (*P* < 0.01), but not relative humidity (*P* = 0.741) (Figure S13). A combination of MAT, MAP and SUN explained 33.0% of the variance in lake TP (F = 90.21, *P* < 0.01). The observed influence of climate on TOC is mediated via the process that affects the carbon exchange between lake ecosystem and the surrounding ecosystems such as limiting the terrestrial productivity^11^. Previous studies have documented the climate controls on C, N and P cycles in the biosphere. Rising temperature not only could stimulate decomposition of peat soils and increase the movement rate of TOC from peat soils to aquatic ecosystem^26^, but also could accelerate the mineralization of OC^2^ and N^5^. MAT were significantly related to microbial N and P^16^. Xu *et al.*^13^ found that MAT and MAP had a significant effect on microbial biomass C, N and P. High rainfall precipitation was expected to lead to the increase in P flush out, and thus maintained the oligotrophic levels in a Mediterranean lake^27^. Mean annual runoff and precipitation have been found to affect OC concentrations^11^. Our report on the global landscape of TOC, TN and TP in lake water would be helpful in making management strategies to keep their cycles in reasonable balance or to minimize their negative effects on the environment.

## Additional Information

**How to cite this article**: Chen, M. *et al.* Global Landscape of Total Organic Carbon, Nitrogen and Phosphorus in Lake Water. *Sci. Rep.* **5**, 15043; doi: 10.1038/srep15043 (2015).

## Supplementary Material

Supplementary Information

## Figures and Tables

**Table 1:** Summarized mean concentrations and storage of TOC, TN, TP and Chl *a* in global lake water.

	TOC	TN	TP	Chl *a*
Concentrations (mg L^−1^)				
Global lakes	5.578 ± 2.8 (5.4325–5.7266)	0.526 ± 2.6 (0.5123–0.5398)	0.014 ± 3.7 (0.0136–0.0144)	0.0066 ± 5.4 (0.0062–0.0070)
Deep vs. shallow lakes				
*Deep lakes*	3.463 ± 3.3 (2.8366–4.2277)	0.611 ± 2.5 (0.5459–0.6845)	0.017 ± 3.5 (0.0153–0.0197)	0.004 ± 3.6 (0.0035–0.0045)
*Shallow lakes*	7.291 ± 2.6 (6.7313–7.8977)	0.893 ± 2.3 (0.8468–0.9408)	0.034 ± 3.3 (0.0318–0.0364)	0.011 ± 3.3 (0.0100–0.0117)
Lakes divided by climate zone				
*North frigid zone*	3.690 ± 2.6 (3.3589–4.0542)	0.322 ± 2.8 (0.2919–0.3550)	0.00797 ± 3.8 (0.0070–0.0090)	nd
*South temperate zone*	nd	nd	0.033 ± 7.0 (0.0248–0.0435)	nd
*North temperate zone*	5.809 ± 2.8 (5.6533–5.9690)	0.560 ± 2.5 (0.5448–0.5748)	0.014 ± 3.5 (0.0136–0.0145)	nd
Storage (Tg)				
Global lakes	984.0	92.8	2.5	1.2

Values in parentheses represent 95% confidence intervals. TOC, total organic carbon; TN, total nitrogen; TP, total phosphorus; Chl *a*, Chlorophyll *a*; nd, not determined.

**Table 2:** Summarized molar TOC:TN, TOC:TP and TN:TP ratios for global lake water and lakes with different trophic states.

	TOC:TN	TOC:TP	TN:TP
Global lakes	21 ± 2.3 (20.64–21.79)	1467 ± 2.9 (1426.92–1508.00)	74 ± 2.5 (72.31–76.02)
Lakes divided by trophic states			
*Oligotrophic*	nd	2185 ± 2.5 (2111.54–2260.48)	129 ± 1.9 (125.37–132.98)
*Mesotrophic*	nd	1387 ± 2.3 (1338.14–1436.81)	71 ± 1.8 (68.77–72.58)
*Eutrophic*	nd	375 ± 2.7 (349.54–403.09)	35 ± 2.7 (33.17–36.90)

Values in parentheses represent 95% confidence intervals. TOC, total organic carbon; TN, total nitrogen; TP, total phosphorus; nd, not determined.
