# Supplementary material for: Global Landscape of Total Organic Carbon, Nitrogen and Phosphorus in Lake Water
Source: Sci Rep. 2015 Oct 19;5:15043. doi: 10.1038/srep15043 (PMC4609951; doi:10.1038/srep15043)
Supplement: Supplementary Information [file srep15043-s1.pdf]

Supporting Information for

Global landscape of total organic carbon, nitrogen and phosphorus in lake water

Ming Chen, Guangming Zeng, * Jiachao Zhang, Piao Xu, Anwei Chen, Lunhui Lu

correspondence to: zgming@hnu.edu.cn

This PDF file includes:

Sources of Data
Tables S1
Figs. S1 to S13

Sources of Data

1. Sobek, S., Tranvik, L.J., Prairie, Y.T., Kortelainen, P. & Cole, J.J. Patterns and regulation of dissolved organic carbon: An analysis of 7,500 widely distributed lakes. *Limnology And Oceanography* **52**, 1208-1219 (2007).
2. Gu, B.H., Schelske, C.L. & Waters, M.N. Patterns and controls of seasonal variability of carbon stable isotopes of particulate organic matter in lakes. *Oecologia* **165**, 1083-1094 (2011).
3. Zan, F., Huo, S., Xi, B., Zhang, J., Liao, H., Wang, Y. & Yeager, K.M. A 60-year sedimentary record of natural and anthropogenic impacts on Lake Chenghai, China. *Journal of Environmental Sciences* **24**, 602-609 (2012).
4. Wang, S.M. & Dou, H.S. Records of Chinese Lakes. *Beijing Science Press, Beijing*, 377–378 (1998).
5. Yamamuro, M. Chemical tracers of sediment organic matter origins in two coastal lagoons. *Journal Of Marine Systems* **26**, 127-134 (2000).
6. Yamamuro, M. & Kanai, Y. A 200-year record of natural and anthropogenic changes in water quality from coastal lagoon sediments of Lake Shinji, Japan. *Chemical Geology* **218**, 51-61 (2005).
7. Vandergucht, D.M., Sereda, J.M., Davies, J.M. & Hudson, J.J. A comparison of phosphorus deficiency indicators with steady state phosphate in lakes. *Water Research* **47**, 1816-1826 (2013).
8. Xu, H., Yang, L.Z., Zhao, G.M., Jiao, J.G., Yin, S.X. & Liu, Z.P. Anthropogenic Impact on Surface Water Quality in Taihu Lake Region, China. *Pedosphere* **19**, 765-778 (2009).
9. Zeng, J., Yang, L.Y., Du, H.W., Xiao, L., Jiang, L.J., Wu, J. & Wang, X.L. Bacterioplankton community structure in a eutrophic lake in relation to water chemistry. *World Journal Of Microbiology & Biotechnology* **25**, 763-772 (2009).
10. Zhang, Y., Zhang, H., Gao, X. & Peng, B. Improved AHP method and its application in lake environmental comprehensive quality evaluation—a case study of Xuanwu Lake, Nanjing, China. *Chinese Journal of Oceanology and Limnology* **25**, 427-433 (2007).
11. Xu, Y., Wang, G., Yang, W. & Li, R. Dynamics of the water bloom-forming Microcystis and its relationship with physicochemical factors in Lake Xuanwu (China). *Environmental Science and Pollution Research* **17**, 1581-1590 (2010).
12. Cornwell, J.C. & Kipphut, G.W. Biogeochemistry of manganese-and iron-rich sediments in Toolik Lake, Alaska. *Hydrobiologia* **240**, 45-59 (1992).
13. Gui, Z.F., Xue, B., Yao, S.C., Zhang, F.J. & Yi, S.H. Catchment erosion and trophic status changes over the past century as recorded in sediments from Wudalianchi Lake, the northernmost volcanic lake in China. *Quaternary International* **282**, 163-170 (2012).
14. Fukushima, T., Aizaki, M. & Muraoka, K. in Sediment/Water Interactions 279-295 (Springer, 1989).
15. Tong, Y., Lin, G.F., Ke, X., Liu, F.P., Zhu, G.W., Gao, G. & Shen, J.H. Comparison of microbial community between two shallow freshwater lakes in middle Yangtze basin, East China. *Chemosphere* **60**, 85-92 (2005).
16. Li, X., Cui, B., Yang, Q., Tian, H., Lan, Y., Wang, T. & Han, Z. Detritus quality controls macrophyte decomposition under different nutrient concentrations in a eutrophic shallow lake, North China. *PloS one* **7**, e42042 (2012).
17. Cui, B., Li, X. & Zhang, K. Classification of hydrological conditions to assess water allocation schemes for Lake Baiyangdian in North China. *Journal of hydrology* **385**, 247-256 (2010).
18. Petrucio, M. & Barbosa, F. Diel variations of phytoplankton and bacterioplankton production rates in four tropical lakes in the middle Rio Doce basin (southeastern Brazil). *Hydrobiologia* **513**, 71-76 (2004).

19. De Souza, D.B., Machado, K.S., Froehner, S., Scapulatempo, C.F. & Bleninger, T. Distribution of< i> n</i>-alkanes in lacustrine sediments from subtropical lake in Brazil. *Chemie der Erde-Geochemistry* **71**, 171-176 (2011).
20. Reche, I., Pulido-Villena, E., Conde-Porcuna, J.M. & Carrillo, P. Photoreactivity of dissolved organic matter from high-mountain lakes of Sierra Nevada, Spain. *Arctic Antarctic And Alpine Research* **33**, 426-434 (2001).
21. Reche, I., Ortega-Retuerta, E., Romera, O., Pulido-Villena, E., Morales-Baquero, R. & Casamayor, E.O. Effect of Saharan dust inputs on bacterial activity and community composition in Mediterranean lakes and reservoirs. *Limnology And Oceanography* **54**, 869-879 (2009).
22. Mladenov, N., Pulido-Villena, E., Morales-Baquero, R., Ortega-Retuerta, E., Sommaruga, R. & Reche, I. Spatiotemporal drivers of dissolved organic matter in high alpine lakes: Role of Saharan dust inputs and bacterial activity. *Journal Of Geophysical Research-Biogeosciences* **113** (2008).
23. Morales-Baquero, R., Pulido-Villena, E. & Reche, I. Atmospheric inputs of phosphorus and nitrogen to the southwest Mediterranean region: Biogeochemical responses of high mountain lakes. *Limnology And Oceanography* **51**, 830-837 (2006).
24. Vrede, K. Effects of inorganic nutrients and zooplankton on the growth of heterotrophic bacterioplankton-enclosure experiments in an oligotrophic clearwater lake. *Aquatic microbial ecology* **18**, 133-144 (1999).
25. Rosel, S., Rychla, A., Wurzbacher, C. & Grossart, H.P. Effects of pollen leaching and microbial degradation on organic carbon and nutrient availability in lake water. *Aquatic Sciences* **74**, 87-99 (2012).
26. Allgaier, M. & Grossart, H.P. Seasonal dynamics and phylogenetic diversity of free-living and particle-associated bacterial communities in four lakes in northeastern Germany. *Aquatic Microbial Ecology* **45**, 115-128 (2006).
27. Burkert, U., Ginzel, G., Babenzien, H. & Koschel, R. The hydrogeology of a catchment area and an artificially divided dystrophic lake—consequences for the limnology of Lake Fuchskuhle. *Biogeochemistry* **71**, 225-246 (2005).
28. Mallin, M.A., Johnson, V.L., Ensign, S.H. & MacPherson, T.A. Factors contributing to hypoxia in rivers, lakes, and streams. *Limnology And Oceanography* **51**, 690-701 (2006).
29. Wu, S., Wang, S., Yang, H., Xie, P., Ni, L. & Xu, J. Field studies on the environmental factors in controlling microcystin production in the subtropical shallow lakes of the Yangtze River. *Bulletin Of Environmental Contamination And Toxicology* **80**, 329-334 (2008).
30. Kim, B., Choi, K., Kim, C., Lee, U.H. & Kim, Y.H. Effects of the summer monsoon on the distribution and loading of organic carbon in a deep reservoir, Lake Soyang, Korea. *Water Research* **34**, 3495-3504 (2000).
31. Hwang, S.-J., Kim, H.-S., Shin, J.-K., Oh, J.-M. & Kong, D.-S. Grazing effects of a freshwater bivalve (Corbicula leana Prime) and large zooplankton on phytoplankton communities in two Korean lakes. *Hydrobiologia* **515**, 161-179 (2004).
32. Choi, K., Kim, B. & Lee, U.H. Characteristics of dissolved organic carbon in three layers of a deep reservoir, Lake Soyang, Korea. *International review of hydrobiology* **86**, 63-76 (2001).
33. Kim, Y. & Kim, B. Application of a 2-dimensional water quality model (CE-QUAL-W2) to the turbidity interflow in a deep reservoir (Lake Soyang, Korea). *Lake and Reservoir Management* **22**, 213-222 (2006).
34. Kim, H.-S., Hwang, S.-J. & Ko, J.-M. Evaluation of Water Quality Variation and Sediment of a

Shallow Artificial Lake (Lake Ilgam) in Located the Metropolitan Area. *Korean Journal of Limnology* **36**, 161-171 (2003).
35. Hwang, S.-J., Kim, H.-S., Park, J.-H. & Kim, B.-H. Shift in nutrient and plankton community in eutrophic lake following introduction of a freshwater bivalve. (2011).
36. Spierenburg, P., Roelofs, J.G.M., Andersen, T.J. & Lotter, A.F. Historical changes in the macrophyte community of a Norwegian softwater lake. *Journal Of Paleolimnology* **44**, 841-853 (2010).
37. Guo, H.Y., Wang, X.R. & Zhu, J.G. Quantification and index of non-point source pollution in Taihu Lake region with GIS. *Environmental Geochemistry And Health* **26**, 147-156 (2004).
38. Qin, B.Q., Xu, P.Z., Wu, Q.L., Luo, L.C. & Zhang, Y.L. Environmental issues of Lake Taihu, China. *Hydrobiologia* **581**, 3-14 (2007).
39. Li, J., Liu, C.Q. & Zhu, Z.Z. Historical eutrophication in Lake Taihu: evidence from biogenic silica and total phosphorus accumulation in sediments from northern part of Lake Taihu. *Environmental Geology* **55**, 1493-1500 (2008).
40. Wu, J.L., Lin, L., Gagan, M.K., Schleser, G.H. & Wang, S.M. Organic matter stable isotope (delta C-13, delta N-15) response to historical eutrophication of lake Taihu, China. *Hydrobiologia* **563**, 19-29 (2006).
41. Kahlert, M. Horizontal variation of biomass and C : N : P ratios of benthic algae in lakes. *Hydrobiologia* **489**, 171-177 (2002).
42. Kenney, W.F., Brenner, M., Curtis, J.H. & Schelske, C.L. Identifying sources of organic matter in sediments of shallow lakes using multiple geochemical variables. *Journal Of Paleolimnology* **44**, 1039-1052 (2010).
43. Chen, W., Chen, Y., Gao, X. & Yoshida, I. Eutrophication of Taihu and its control. *Agri. Engin. J* **6**, 109-120 (1997).
44. Wu, Q.L., Chen, Y., Xu, K., Liu, Z. & Hahn, M.W. Intra-habitat heterogeneity of microbial food web structure under the regime of eutrophication and sediment resuspension in the large subtropical shallow Lake Taihu, China. *Hydrobiologia* **581**, 241-254 (2007).
45. Chen, Y.W., Qin, B.Q., Teubner, K. & Dokulil, M.T. Long-term dynamics of phytoplankton assemblages: Microcystis-domination in Lake Taihu, a large shallow lake in China. *Journal Of Plankton Research* **25**, 445-453 (2003).
46. Vrede, T. & Tranvik, L.J. Iron constraints on planktonic primary production in oligotrophic lakes. *Ecosystems* **9**, 1094-1105 (2006).
47. Lim, D.S., Douglas, M.S. & Smol, J.P. Limnology of 46 lakes and ponds on Banks Island, NWT, Canadian Arctic Archipelago. *Hydrobiologia* **545**, 11-32 (2005).
48. 2. Hehmann, A., Krienitz, L. & Koschel, R. Long-term phytoplankton changes in an artificially divided, top-down manipulated humic lake. *Hydrobiologia* **448**, 83-96 (2001).
49. Qu, W.C., Dickman, M. & Wang, S.M. Multivariate analysis of heavy metal and nutrient concentrations in sediments of Taihu Lake, China. *Hydrobiologia* **450**, 83-89 (2001).
50. Manny, B.A., Johnson, W.C. & Wetzel, R.G. Nutrient additions by waterfowl to lakes and reservoirs: predicting their effects on productivity and water quality. *Hydrobiologia* **279-280**, 121-132 (1994).
51. Vrede, K. Nutrient and temperature limitation of bacterioplankton growth in temperate lakes. *Microb Ecol* **49**, 245-56 (2005).
52. Beamud, S.G., Diaz, M.M. & Pedrozo, F.L. Nutrient limitation of phytoplankton in a naturally acidic lake (Lake Caviahue, Argentina). *Limnology* **11**, 103-113 (2010).

53. Rapacioli, R. The Lake Caviahue and its Basin. Technical Report of the EPAS Government Office, Province of Neuquén, Argentina. 1–72 (1985).
54. Pedrozo, F., Kelly, L., Diaz, M., Temporetti, P., Baffico, G., Kringel, R., Friese, K., Mages, M., Geller, W. & Woelfl, S. First results on the water chemistry, algae and trophic status of an Andean acidic lake system of volcanic origin in Patagonia (Lake Caviahue). *Hydrobiologia* **452**, 129-137 (2001).
55. Pedrozo, F.L., Temporetti, P.F., Beamud, G. & Diaz, M.M. Volcanic nutrient inputs and trophic state of Lake Caviahue, Patagonia, Argentina. *Journal Of Volcanology And Geothermal Research* **178**, 205-212 (2008).
56. Håkanson, L. On the relationship between lake trophic level and lake sediments. *Water research* **18**, 303-314 (1984).
57. Nowlin, H. Phosphorus dynamics in coastal and inland lakes and reservoirs of British Columbia. *Ph.D. thesis, Univ.of Victoria* (2003).
58. Davies, J.M. Linking ecology and management of water quality: The distribution and growth of phytoplankton in coastal lakes of British Columbia. *Ph.D. thesis, Univ. of Victoria* (2004).
59. Grey, J., Jones, R.I. & Sleep, D. Seasonal changes in the importance of the source of organic matter to the diet of zooplankton in Loch Ness, as indicated by stable isotope analysis. *Limnology And Oceanography* **46**, 505-513 (2001).
60. Zeng, Q.F., Kong, F.X., Zhang, E.L., Tan, X. & Wu, X.D. Seasonality of stable carbon and nitrogen isotopes within the pelagic food web of Taihu Lake. *Annales De Limnologie-International Journal Of Limnology* **44**, 1-6 (2008).
61. Matthews, B. & Mazumder, A. Habitat specialization and the exploitation of allochthonous carbon by zooplankton. *Ecology* **87**, 2800-2812 (2006).
62. Moschen, R., Lucke, A., Parplies, J. & Schleser, G.H. Controls on the seasonal and interannual dynamics of organic matter stable carbon isotopes in mesotrophic Lake Holzmaar, Germany. *Limnology And Oceanography* **54**, 194-209 (2009).
63. Pulido-Villena, E., Reche, I. & Morales-Baquero, R. Food web reliance on allochthonous carbon in two high mountain lakes with contrasting catchments: a stable isotope approach. *Canadian Journal Of Fisheries And Aquatic Sciences* **62**, 2640-2648 (2005).
64. Takamura, N., Nakagawa, M., Nakajima, H., Wakana, I., Ito, T. & Igarashi, S. Features of Lake Takkobu [Japan] ecosystem assessed by natural abundance of 13C and 15N. *Japanese Journal of Limnology* **68**, 169-186 (2007).
65. Gu, B.H., Schelske, C.L. & Hodell, D.A. Extreme C-13 enrichments in a shallow hypereutrophic lake: Implications for carbon cycling. *Limnology And Oceanography* **49**, 1152-1159 (2004).
66. Gu, B.H., Chapman, A.D. & Schelske, C.L. Factors controlling seasonal variations in stable isotope composition of particulate organic matter in a soft water eutrophic lake. *Limnology And Oceanography* **51**, 2837-2848 (2006).
67. Gu, B., Schell, D.M. & Alexander, V. Stable carbon and nitrogen isotopic analysis of the plankton food web in a subarctic lake. *Canadian Journal of Fisheries and Aquatic Sciences* **51**, 1338-1344 (1994).
68. Gu, B. & Schelske, L. Temporal and spatial variations in phytoplankton carbon isotopes in a polymictic subtropical lake. *Journal of plankton research* **18**, 2081-2092 (1996).
69. Matthews, B. & Mazumder, A. Temporal variation in body composition (C : N) helps explain seasonal patterns of zooplankton delta C-13. *Freshwater Biology* **50**, 502-515 (2005).
70. SIMONA, M. Winter and spring mixing depths affect the trophic status and composition of

phytoplankton in the northern meromictic basin of Lake Lugano. *Journal of Limnology* **62**, 190-206 (2003).
71. Takamura, N., Kadono, Y., Fukushima, M., Nakagawa, M. & Kim, B.H.O. Effects of aquatic macrophytes on water quality and phytoplankton communities in shallow lakes. *Ecological Research* **18**, 381-395 (2003).
72. Xu, J., Xie, P., Zhang, M. & Yang, H. Variation in stable isotope signatures of seston and a zooplanktivorous fish in a eutrophic Chinese lake. *Hydrobiologia* **541**, 215-220 (2005).
73. Yoshioka, T., Wada, E. & Hayashi, H. A stable isotope study on seasonal food web dynamics in a eutrophic lake. *Ecology*, 835-846 (1994).
74. Owen, J.S., Mitchell, M.J. & Michener, R.H. Stable nitrogen and carbon isotonic composition of seston and sediment in two Adirondack lakes. *Can J Fish Aquat Sci* **56**, 2186-2192 (1999).
75. SyvÄRanta, J., Haemaelaeinen, H. & Jones, R.I. Within ‐ lake variability in carbon and nitrogen stable isotope signatures. *Freshwater Biology* **51**, 1090-1102 (2006).
76. Vuorio, K., Meili, M. & Sarvala, J. Taxon-specific variation in the stable isotopic signatures (delta C-13 and delta N-15) of lake phytoplankton. *Freshwater Biology* **51**, 807-822 (2006).
77. Hollander, D.J. & McKenzie, J.A. CO2 control on carbon-isotope fractionation during aqueous photosynthesis: A paleo-pCO2 barometer. *Geology* **19**, 929-932 (1991).
78. Marotta, H., Duarte, C.M., Sobek, S. & Enrich ‐ Prast, A. Large CO2 disequilibria in tropical lakes. *Global biogeochemical cycles* **23** (2009).
79. Leggett, M., Servos, M., Hesslein, R., Johannsson, O., Millard, E. & Dixon, D. Biogeochemical influences on the carbon isotope signatures of Lake Ontario biota. *Canadian Journal of Fisheries and Aquatic Sciences* **56**, 2211-2218 (1999).
80. McCabe, B. The dynamics of 13C in several New Zealand lakes. *PhD Thesis, University of Waikato* (1985).
81. Davies, J.M., Hesslein, R.H., Kelly, C.A. & Hecky, R.E. PCO2 method for measuring photosynthesis and respiration in freshwater lakes. *Journal Of Plankton Research* **25**, 385-395 (2003).
82. Guildford, S., Bootsma, H., Fee, E., Hecky, R. & Patterson, G. Phytoplankton nutrient status and mean water column irradiance in Lakes Malawi and Superior. *Aquatic Ecosystem Health & Management* **3**, 35-45 (2000).
83. Hecky, R. A biogeochemical comparison of Lakes Superior and Malawi and the limnological consequences of an endless summer. *Aquatic Ecosystem Health & Management* **3**, 23-33 (2000).
84. Zan, F., Huo, S., Xi, B., Li, Q., Liao, H. & Zhang, J. Phosphorus distribution in the sediments of a shallow eutrophic lake, Lake Chaohu, China. *Environmental Earth Sciences* **62**, 1643-1653 (2011).
85. Xu, M.Q., Cao, H., Xie, P., Deng, D.G., Feng, W.S. & Xu, H. The temporal and spatial distribution, composition and abundance of Protozoa in Chaohu Lake, China: Relationship with eutrophication. *European Journal Of Protistology* **41**, 183-192 (2005).
86. Xu, F. Exergy and structural exergy as ecological indicators for the development state of the Lake Chaohu ecosystem. *Ecological Modelling* **99**, 41-49 (1997).
87. Viner, A. & Smith, I. Geographical, historical and physical aspects of Lake George. *Proceedings of the Royal Society of London. Series B. Biological Sciences* **184**, 235-270 (1973).
88. Poste, A.E., Hecky, R.E. & Guildford, S.J. Phosphorus enrichment and carbon depletion contribute to high Microcystis biomass and microcystin concentrations in Ugandan lakes. *Limnology And Oceanography* **58**, 1075-1088 (2013).
89. Melack, J. Morphometric, physical and chemical features of the volcanic crater lakes of western

Uganda. *Archiv fur Hydrobiologie* **84**, 430-453 (1978).

90. Nyakoojo, C. & Byarujali, S.M. An ecological study of two shallow, equatorial lakes: Lake Mburo and Lake Kachera, Uganda. *African Journal Of Ecology* **48**, 860-864 (2010).
91. Haande, S., Rohrlack, T., Semyalo, R.P., Brettum, P., Edvardsen, B., Lyche-Solheim, A., Sorensen, K. & Larsson, P. Phytoplankton dynamics and cyanobacterial dominance in Murchison Bay of Lake Victoria (Uganda) in relation to environmental conditions. *Limnologica* **41**, 20-29 (2011).
92. Chapman, L.J., Chapman, C.A., Crisman, T.L. & Nordlie, F.G. Dissolved oxygen and thermal regimes of a Ugandan crater lake. *Hydrobiologia* **385**, 201-211 (1998).
93. Poste, A.E., Hecky, R.E. & Guildford, S.J. Evaluating Microcystin Exposure Risk through Fish Consumption. *Environmental Science & Technology* **45**, 5806-5811 (2011).
94. Okello, W. & Kurmayer, R. Seasonal development of cyanobacteria and microcystin production in Ugandan freshwater lakes. *Lakes & Reservoirs: Research & Management* **16**, 123-135 (2011).
95. Graneli, W., Bertilsson, S. & Philibert, A. Phosphorus limitation of bacterial growth in high Arctic lakes and ponds. *Aquatic Sciences* **66**, 430-439 (2004).
96. Michelutti, N., Douglas, M.S.V., Lean, D.R.S. & Smol, J.P. Physical and chemical limnology of 34 ultra-oligotrophic lakes and ponds near Wynniatt Bay, Victoria Island, Arctic Canada. *Hydrobiologia* **482**, 1-13 (2002).
97. Steinhart, G.S., Likens, G.E. & Soto, D. Physiological indicators of nutrient deficiency in phytoplankton in southern Chilean lakes. *Hydrobiologia* **489**, 21-27 (2002).
98. Peel, M.C., Finlayson, B.L. & McMahon, T.A. Updated world map of the Koppen-Geiger climate classification. *Hydrology And Earth System Sciences* **11**, 1633-1644 (2007).
99. Silvoso, J., Izaguirre, I. & Allende, L. Picoplankton structure in clear and turbid eutrophic shallow lakes: A seasonal study. *Limnologica* **41**, 181-190 (2011).
100. Overmann, J., Beatty, J.T. & Hall, K.J. Purple sulfur bacteria control the growth of aerobic heterotrophic bacterioplankton in a meromictic salt lake. *Applied and environmental microbiology* **62**, 3251-3258 (1996).
101. Coolen, M.J. & Overmann, J. Analysis of subfossil molecular remains of purple sulfur bacteria in a lake sediment. *Applied and environmental microbiology* **64**, 4513-4521 (1998).
102. Overmann, J., Sandmann, G., Hall, K.J. & Northcote, T.G. Fossil carotenoids and paleolimnology of meromictic Mahoney Lake, British Columbia, Canada. *Aquatic sciences* **55**, 31-39 (1993).
103. Heinrichs, M.L., Wilson, S.E., Walker, I.R., Smol, J.P., Mathewes, R.W. & Hall, K.J. Midge-and diatom-based palaeosalinity reconstructions for Mahoney lake, Okanagan Valley, British Columbia, Canada. *International Journal of Salt Lake Research* **6**, 249-267 (1997).
104. Overmann, J. & Pfennig, N. Bouyancy regulation and aggregate formation in< i> Amoebobacter purpureus</i> from Mahoney Lake. *FEMS Microbiology Letters* **101**, 67-79 (1992).
105. Overmann, J., Beatty, J.T., Hall, K.J., Pfennig, N. & Northcote, T.G. Characterization of a dense, purple sulfur bacterial layer in a meromictic salt lake. *Limnology and oceanography*, 846-859 (1991).
106. Malley, D.F., Williams, P.C. & Stainton, M.P. Rapid measurement of suspended C, N, and P from precambrian shield lakes using near-infrared reflectance spectroscopy. *Water Research* **30**, 1325-1332 (1996).
107. Vrede, K. Regulation of bacterioplankton production and biomass in an oligotrophic cleanvater lake—the importance of the phytoplankton community. *Journal of plankton research* **18**, 1009-1032 (1996).

108. Kenney, W.F., Waters, M.N., Schelske, C.L. & Brenner, M. Sediment records of phosphorus-driven shifts to phytoplankton dominance in shallow Florida lakes. *Journal Of Paleolimnology* **27**, 367-377 (2002).
109. Bachmann, R.W., Hoyer, M.V. & Canfield Jr, D.E. Internal heterotrophy following the switch from macrophytes to algae in Lake Apopka, Florida. *Hydrobiologia* **418**, 217-227 (2000).
110. Schelske, C.L., Lowe, E.F., Battoe, L.E., Brenner, M., Coveney, M.F. & Kenney, W.F. Abrupt biological response to hydrologic and land-use changes in Lake Apopka, Florida, USA. *AMBIO: A Journal of the Human Environment* **34**, 192-198 (2005).
111. Silliman, J.E. & Schelske, C.L. Saturated hydrocarbons in the sediments of Lake Apopka, Florida. *Organic Geochemistry* **34**, 253-260 (2003).
112. Brenner, M., Whitmore, T.J., Lasi, M.A., Cable, J.E. & Cable, P.H. A multi-proxy trophic state reconstruction for shallow Orange Lake, Florida, USA: possible influence of macrophytes on limnetic nutrient concentrations. *Journal of Paleolimnology* **21**, 215-233 (1999).
113. Stets, E.G. & Cotner, J.B. The influence of dissolved organic carbon on bacterial phosphorus uptake and bacteria phytoplankton dynamics in two Minnesota lakes. *Limnology And Oceanography* **53**, 137-147 (2008).
114. Prairie, Y.T., Bird, D.F. & Cole, J.J. The summer metabolic balance in the epilimnion of southeastern Quebec lakes. *Limnology and Oceanography* **47**, 316-321 (2002).
115. Naddafi, R., Goedkoop, W., Grandin, U. & Eklov, P. Variation in tissue stoichiometry and condition index of zebra mussels in invaded Swedish lakes. *Biological Invasions* **14**, 2117-2131 (2012).
116. Hakulinen, R., Kahkonen, M.A. & Salkinoja-Salonen, M. Vertical distribution of sediment enzyme activities involved in the cycling of carbon, nitrogen, phosphorus and sulphur in three boreal rural lakes. *Water Research* **39**, 2319-2326 (2005).
117. Suominen, K.P., Jaakkola, T., Elomaa, E., Hakulinen, R. & Salkinoja-Salonen, M.S. Sediment accumulation of organic halogens in pristine forest lakes. *Environmental Science and Pollution Research* **4**, 21-30 (1997).
118. Ao, H., Wu, C., Xiong, X., Jing, L., Huang, X., Zhang, K. & Liu, J. Water and sediment quality in Qinghai Lake, China: a revisit after half a century. *Environmental monitoring and assessment*, 1-13 (2013).
119. Xu, H., Hou, Z., An, Z., Liu, X. & Dong, J. Major ion chemistry of waters in Lake Qinghai catchments, NE Qinghai-Tibet plateau, China. *Quaternary International* **212**, 35-43 (2010).
120. Liu, W., Liu, Z., Wang, H., He, Y., Wang, Z. & Xu, L. Salinity control on long-chain alkenone distributions in lake surface waters and sediments of the northern Qinghai-Tibetan Plateau, China. *Geochimica et Cosmochimica Acta* **75**, 1693-1703 (2011).
121. Li, X., Liu, W., Zhang, L. & Sun, Z. Distribution of Recent ostracod species in the Lake Qinghai area in northwestern China and its ecological significance. *Ecological indicators* **10**, 880-890 (2010).
122. Henderson, A.C. & Holmes, J.A. Palaeolimnological evidence for environmental change over the past millennium from Lake Qinghai sediments: a review and future research prospective. *Quaternary International* **194**, 134-147 (2009).
123. Henderson, A.C., Holmes, J.A. & Leng, M.J. Late Holocene isotope hydrology of Lake Qinghai, NE Tibetan Plateau: effective moisture variability and atmospheric circulation changes. *Quaternary Science Reviews* **29**, 2215-2223 (2010).
124. Zhao, Y., Yu, Z., Chen, F., Liu, X. & Ito, E. Sensitive response of desert vegetation to moisture change based on a near-annual resolution pollen record from Gahai Lake in the Qaidam Basin,

northwest China. *Global and Planetary Change* **62**, 107-114 (2008).
125. Fan, Q., Lai, Z., Long, H., Sun, Y. & Liu, X. OSL chronology for lacustrine sediments recording high stands of Gahai Lake in Qaidam Basin, northeastern Qinghai–Tibetan Plateau. *Quaternary Geochronology* **5**, 223-227 (2010).
126. He, Y., Liu, W., Zhao, C., Wang, Z., Wang, H., Liu, Y., Qin, X., Hu, Q., An, Z. & Liu, Z. Solar influenced late Holocene temperature changes on the northern Tibetan Plateau. *Chinese Science Bulletin* **58**, 1053-1059 (2013).
127. Li, W., Cao, T., Ni, L.Y., Zhang, X.L., Zhu, G.R. & Xie, P. Effects of water depth on carbon, nitrogen and phosphorus stoichiometry of five submersed macrophytes in an in situ experiment. *Ecological Engineering* **61**, 358-365 (2013).
128. Wu, Q.L., Zwart, G., Schauer, M., Kamst-van Agterveld, M.P. & Hahn, M.W. Bacterioplankton community composition along a salinity gradient of sixteen high-mountain lakes located on the Tibetan Plateau, China. *Applied and environmental microbiology* **72**, 5478-5485 (2006).
129. Woodward, C.A., Potito, A.P. & Beilman, D.W. Carbon and nitrogen stable isotope ratios in surface sediments from lakes of western Ireland: implications for inferring past lake productivity and nitrogen loading. *Journal Of Paleolimnology* **47**, 167-184 (2012).
130. Prepas, E. & Trimbee, A. Evaluation of indicators of nitrogen limitation in deep prairie lakes with laboratory bioassays and limnocorrals. *Hydrobiologia* **159**, 269-276 (1988).
131. Finlay, K., Leavitt, P.R., Wissel, B. & Prairie, Y.T. Regulation of spatial and temporal variability of carbon flux in six hard-water lakes of the northern Great Plains. *Limnology And Oceanography* **54**, 2553-2564 (2009).
132. Avramidis, P., Samiotis, A., Kalimani, E., Papoulis, D., Lampropoulou, P. & Bekiari, V. Sediment characteristics and water physicochemical parameters of the Lysimachia Lake, Western Greece. *Environmental Earth Sciences* **70**, 383-392 (2013).
133. Yuan, H.Z., An, S.Q., Shen, J. & Liu, E.F. The characteristic and environmental pollution records of phosphorus species in different trophic regions of Taihu Lake, China. *Environmental Earth Sciences* **71**, 783-792 (2014).
134. Åberg, J., Bergström, A.-K., Algesten, G., Söderback, K. & Jansson, M. A comparison of the carbon balances of a natural lake (L. Örträsket) and a hydroelectric reservoir (L. Skinnmuddselet) in northern Sweden. *Water Research* **38**, 531-538 (2004).
135. Page, M., Trustrum, N. & DeRose, R. A high resolution record of storm-induced erosion from lake sediments, New Zealand. *Journal of Paleolimnology* **11**, 333-348 (1994).
136. Orpin, A., Carter, L., Page, M., Cochran, U., Trustrum, N., Gomez, B., Palmer, A., Mildenhall, D., Rogers, K. & Brackley, H. Holocene sedimentary record from Lake Tutira: A template for upland watershed erosion proximal to the Waipaoa Sedimentary System, northeastern New Zealand. *Marine Geology* **270**, 11-29 (2010).
137. Eden, D., Froggatt, P., Trustrum, N. & Page, M. A multiple - source Holocene tephra sequence from Lake Tutira, Hawke's Bay, New Zealand. *New Zealand journal of geology and geophysics* **36**, 233-242 (1993).
138. Zhang, J., Jørgensen, S.E., Tan, C.O. & Beklioglu, M. A structurally dynamic modelling—Lake Mogan, Turkey as a case study. *Ecological Modelling* **164**, 103-120 (2003).
139. Suurkuukka, H., Meissner, K.K. & Muotka, T. Species turnover in lake littorals: spatial and temporal variation of benthic macroinvertebrate diversity and community composition. *Diversity and Distributions* **18**, 931-941 (2012).

140. Ekholm, P. & Mitikka, S. Agricultural lakes in Finland: current water quality and trends. *Environmental Monitoring and Assessment* **116**, 111-135 (2006).
141. Lehours, A.-C., Bardot, C., Thenot, A., Debroas, D. & Fonty, G. Anaerobic microbial communities in Lake Pavin, a unique meromictic lake in France. *Applied and environmental microbiology* **71**, 7389-7400 (2005).
142. Díaz, P., Gerrero, M., Alcorlo, P., Baltanás, A., Florin, M. & Montes, C. Anthropogenic perturbations to the trophic structure in a permanent hypersaline shallow lake: La Salada de Chiprana (north-eastern Spain). *International Journal of Salt Lake Research* **7**, 187-210 (1998).
143. Jonkers, H.M., Ludwig, R., Wit, R., Pringault, O., Muyzer, G., Niemann, H., Finke, N. & Beer, D. Structural and functional analysis of a microbial mat ecosystem from a unique permanent hypersaline inland lake:'La Salada de Chiprana'(NE Spain). *FEMS microbiology ecology* **44**, 175-189 (2003).
144. de Wit, R., Falcon, L.I. & Charpy-Roubaud, C. Heterotrophic dinitrogen fixation (acetylene reduction) in phosphate-fertilised Microcoleus chthonoplastes microbial mat from the hypersaline inland lake 'la Salada de Chiprana'(NE Spain). *Hydrobiologia* **534**, 245-253 (2005).
145. Pajunen, H. Carbon in Finnish lake sediments (Geol. survey pf Finland2000 (оборот тит. л. 2001) Espoo, 2000).
146. Gantidis, N., Pervolarakis, M. & Fytianos, K. Assessment of the quality characteristics of two lakes (Koronia and Volvi) of N. Greece. *Environmental monitoring and assessment* **125**, 175-181 (2007).
147. 16. Mustapha, K. Biochemical oxygen demand in Malta Lake, Poznan, Poland. (2010).
148. 17. Vreca, P. & Muri, G. Changes in accumulation of organic matter and stable carbon and nitrogen isotopes in sediments of two Slovenian mountain lakes(Lake Ledvica and Lake Planina), induced by eutrophication changes. *Limnology and oceanography* **51**, 781-790 (2006).
149. 18. Sachse, A., Babenzien, D., Ginzel, G., Gelbrecht, J. & Steinberg, C. Characterization of dissolved organic carbon (DOC) in a dystrophic lake and an adjacent fen. *Biogeochemistry* **54**, 279-296 (2001).
150. 19. Diaz, M., Pedrozo, F., Reynolds, C. & Temporetti, P. Chemical composition and the nitrogen-regulated trophic state of Patagonian lakes. *Limnologica-Ecology and Management of Inland Waters* **37**, 17-27 (2007).
151. Åberg, J., Bergström, A.-K., Algesten, G., Söderback, K. & Jansson, M. A comparison of the carbon balances of a natural lake (L. Örträsket) and a hydroelectric reservoir (L. Skinnmuddselet) in northern Sweden. *Water Research* **38**, 531-538 (2004).
152. Page, M., Trustrum, N. & DeRose, R. A high resolution record of storm-induced erosion from lake sediments, New Zealand. *Journal of Paleolimnology* **11**, 333-348 (1994).
153. Orpin, A., Carter, L., Page, M., Cochran, U., Trustrum, N., Gomez, B., Palmer, A., Mildenhall, D., Rogers, K. & Brackley, H. Holocene sedimentary record from Lake Tutira: A template for upland watershed erosion proximal to the Waipaoa Sedimentary System, northeastern New Zealand. *Marine Geology* **270**, 11-29 (2010).
154. Eden, D., Froggatt, P., Trustrum, N. & Page, M. A multiple‐source Holocene tephra sequence from Lake Tutira, Hawke's Bay, New Zealand. *New Zealand journal of geology and geophysics* **36**, 233-242 (1993).
155. Zhang, J., Jørgensen, S.E., Tan, C.O. & Beklioglu, M. A structurally dynamic modelling—Lake Mogan, Turkey as a case study. *Ecological Modelling* **164**, 103-120 (2003).
156. Suurkuukka, H., Meissner, K.K. & Muotka, T. Species turnover in lake littorals: spatial and

temporal variation of benthic macroinvertebrate diversity and community composition. *Diversity and Distributions* **18**, 931-941 (2012).

157. Ekholm, P. & Mitikka, S. Agricultural lakes in Finland: current water quality and trends. *Environmental Monitoring and Assessment* **116**, 111-135 (2006).
158. Lehours, A.-C., Bardot, C., Thenot, A., Debroas, D. & Fonty, G. Anaerobic microbial communities in Lake Pavin, a unique meromictic lake in France. *Applied and environmental microbiology* **71**, 7389-7400 (2005).
159. Díaz, P., Gerrero, M., Alcorlo, P., Baltanás, A., Florin, M. & Montes, C. Anthropogenic perturbations to the trophic structure in a permanent hypersaline shallow lake: La Salada de Chiprana (north-eastern Spain). *International Journal of Salt Lake Research* **7**, 187-210 (1998).
160. Jonkers, H.M., Ludwig, R., Wit, R., Pringault, O., Muyzer, G., Niemann, H., Finke, N. & Beer, D. Structural and functional analysis of a microbial mat ecosystem from a unique permanent hypersaline inland lake:'La Salada de Chiprana'(NE Spain). *FEMS microbiology ecology* **44**, 175-189 (2003).
161. de Wit, R., Falcon, L.I. & Charpy-Roubaud, C. Heterotrophic dinitrogen fixation (acetylene reduction) in phosphate-fertilised Microcoleus chthonoplastes microbial mat from the hypersaline inland lake 'la Salada de Chiprana'(NE Spain). *Hydrobiologia* **534**, 245-253 (2005).
162. Pajunen, H. Carbon in Finnish lake sediments (Geol. survey pf Finland2000 (оборот тит. л. 2001) Espoo, 2000).
163. Hallare, A., Kosmehl, T., Schulze, T., Hollert, H., Köhler, H.-R. & Triebskorn, R. Assessing contamination levels of Laguna Lake sediments (Philippines) using a contact assay with zebrafish (< i> Danio rerio</i>) embryos. *Science of the total environment* **347**, 254-271 (2005).
164. Gantidis, N., Pervolarakis, M. & Fytianos, K. Assessment of the quality characteristics of two lakes (Koronia and Volvi) of N. Greece. *Environmental monitoring and assessment* **125**, 175-181 (2007).
165. Mustapha, K. Biochemical oxygen demand in Malta Lake, Poznan, Poland. (2010).
166. Vreca, P. & Muri, G. Changes in accumulation of organic matter and stable carbon and nitrogen isotopes in sediments of two Slovenian mountain lakes(Lake Ledvica and Lake Planina), induced by eutrophication changes. *Limnology and oceanography* **51**, 781-790 (2006).
167. Sachse, A., Babenzien, D., Ginzel, G., Gelbrecht, J. & Steinberg, C. Characterization of dissolved organic carbon (DOC) in a dystrophic lake and an adjacent fen. *Biogeochemistry* **54**, 279-296 (2001).
168. Diaz, M., Pedrozo, F., Reynolds, C. & Temporetti, P. Chemical composition and the nitrogen-regulated trophic state of Patagonian lakes. *Limnologica-Ecology and Management of Inland Waters* **37**, 17-27 (2007).
169. Albay, M. & Akcaalan, R. Comparative study of periphyton colonisation on common reed (Phragmites australis) and artificial substrate in a shallow lake, Manyas, Turkey. *Hydrobiologia* **506**, 531-540 (2003).
170. Burns, N., Deely, J., Hall, J. & Safi, K. Comparing past and present trophic states of seven Central Volcanic Plateau lakes, New Zealand. *New Zealand Journal of Marine and Freshwater Research* **31**, 71-87 (1997).
171. Muri, G. Physico-chemical characteristics of lake water in 14 Slovenian mountain lakes. *ACTA CHIMICA SLOVENICA.* **51**, 257-272 (2004).
172. Muri, G., Čermelj, B., Jaćimović, R., Skaberne, D., Šmuc, A., Šturm, M.B., Turšič, J. & Vreča, P. Consequences of anthropogenic activity for two remote alpine lakes in NW Slovenia as tracked

by sediment geochemistry. *Journal of Paleolimnology* **50**, 457-470 (2013).
173. Jaani, A. The location, size and general characterization of Lake Peipsi and its catchment area. *Lake Peipsi. meteorology, hydrology, hydrochemistry*, 10-17 (2001).
174. Nõges, T., Tuvikene, L. & Nõges, P. Contemporary trends of temperature, nutrient loading, and water quality in large Lakes Peipsi and Võrtsjärv, Estonia. *Aquatic Ecosystem Health & Management* **13**, 143-153 (2010).
175. Mhlanga, L., Mhlanga, W., Mwera, P. & Tendaupenyu, P. Could phosphorus concentrations increase with the development of cage culture in Lake Kariba, Zimbabwe? *African Journal of Aquatic Science*, 1-3 (2014).
176. Vidal, L. & Kruk, C. Cylindrospermopsis raciborskii (Cyanobacteria) extends its distribution to Latitude 34 53′ S: taxonomical and ecological features in Uruguayan eutrophic lakes. *Pan-American Journal of Aquatic Sciences* **3**, 142-151 (2008).
177. Kruk, C., RODRÍGUEZ‐GALLEGO, L., Meerhoff, M., Quintans, F., Lacerot, G., Mazzeo, N., Scasso, F., Paggi, J.C., Peeters, E.T. & Marten, S. Determinants of biodiversity in subtropical shallow lakes (Atlantic coast, Uruguay). *Freshwater Biology* **54**, 2628-2641 (2009).
178. Baier, J., Lücke, A., Negendank, J.F., Schleser, G.-H. & Zolitschka, B. Diatom and geochemical evidence of mid-to late Holocene climatic changes at Lake Holzmaar, West-Eifel (Germany). *Quaternary international* **113**, 81-96 (2004).
179. Brodersen, K.P. & Anderson, N. Distribution of chironomids (Diptera) in low arctic West Greenland lakes: trophic conditions, temperature and environmental reconstruction. *Freshwater Biology* **47**, 1137-1157 (2002).
180. Bigler, C., Heiri, O., Krskova, R., Lotter, A.F. & Sturm, M. Distribution of diatoms, chironomids and cladocera in surface sediments of thirty mountain lakes in south-eastern Switzerland. *Aquatic Sciences* **68**, 154-171 (2006).
181. Istvánovics, V., Honti, M., Kovács, Á. & Osztoics, A. Distribution of submerged macrophytes along environmental gradients in large, shallow Lake Balaton (Hungary). *Aquatic Botany* **88**, 317-330 (2008).
182. Kopáček, J., StuchĺÍik, E., Vyhnálekl, V. & Závodský, D. Concentration of nutrients in selected lakes in the High Tatra Mountains, Slovakia: Effect of season and watershed. *Hydrobiologia* **319**, 47-55 (1996).
183. Søndergaard, M., Kristensen, P. & Jeppesen, E. Phosphorus release from resuspended sediment in the shallow and wind-exposed Lake Arresø, Denmark. *Hydrobiologia* **228**, 91-99 (1992).
184. Siong, K. & Asaeda, T. Effect of magnesium on charophytes calcification: implications for phosphorus speciation stored in biomass and sediment in Myall Lake (Australia). *Hydrobiologia* **632**, 247-259 (2009).
185. Riis, T. & Hawes, I. Effect of wave exposure on vegetation abundance, richness and depth distribution of shallow water plants in a New Zealand lake. *Freshwater Biology* **48**, 75-87 (2003).
186. Sosnovsky, A. & Quirós, R. Effects of fish manipulation on the plankton community in small hypertrophic lakes from the Pampa Plain (Argentina). *Limnologica-Ecology and Management of Inland Waters* **39**, 219-229 (2009).
187. Cogels, F., Fraboulet-Jussila, S. & Varis, O. Multipurpose use and water quality challenges in Lac de Guiers (Senegal). *Water Science & Technology* **44**, 35-46 (2001).
188. Kâ, S., Pagano, M., Bâ, N., Bouvy, M., Leboulanger, C., Arfi, R., Thiaw, O.T., Ndour, E.H.M., Corbin, D. & Defaye, D. Zooplankton distribution related to environmental factors and phytoplankton in a

shallow tropical lake (Lake Guiers, Senegal, West Africa). *International review of hydrobiology* **91**, 389-405 (2006).

189. Kâ, S., Bouvy, M., Sané, S., Ba, N., Arfi, R., Thiaw, O.T. & Pagano, M. Zooplankton communities in the shallow lake Guiers (Senegal, West Africa). *International Review of Hydrobiology* **96**, 405-424 (2011).
190. Qumos, R. Relationships between air temperature, depth, nutrients and chlorophyll in 103 Argentinian lakes. (1988).
191. Hart, R. South African coastal lakes.
192. Chardhry, P., Sharma, M., Bhargava, R., Kumar, S. & Dadhwal, P. Water quality assessment of Sukhna Lake of Chandigarh city of India. *Hydro Nepal: Journal of Water, Energy and Environment* **12**, 26-31 (2013).
193. Whitmore, T., Brenner, M., Jiang, Z., Curtis, J., Moore, A., Engstrom, D. & Wu, Y. Water quality and sediment geochemistry in lakes of Yunnan Province, southern China. *Environmental geology* **32**, 45-55 (1997).
194. Nurminen, L. Macrophyte species composition reflecting water quality changes in adjacent water bodies of lake Hiidenvesi, SW Finland. *Annales Botanici Fennici* **40**, 199-208 (2003).
195. Rosén, P. Total organic carbon (TOC) of lake water during the Holocene inferred from lake sediments and near-infrared spectroscopy (NIRS) in eight lakes from northern Sweden. *Biogeochemistry* **76**, 503-516 (2005).
196. Kurun, A., Balkıs, N., Erkan, M., Balkıs, H., Aksu, A. & Erşan, M.S. Total metal levels in crayfish Astacus leptodactylus (Eschscholtz, 1823), and surface sediments in Lake Terkos, Turkey. *Environmental monitoring and assessment* **169**, 385-395 (2010).
197. Rogora, M., Massaferro, J., Marchetto, A., Tartari, G. & MOSELLO, R. The water chemistry of some shallow lakes in Northern Patagonia and their nitrogen status in comparison with remote lakes in different regions of the globe. *Journal of Limnology* **67**, 75-86 (2008).
198. Simčič, T. The role of plankton, zoobenthos, and sediment in organic matter degradation in oligotrophic and eutrophic mountain lakes. *Hydrobiologia* **532**, 69-79 (2005).
199. Tadesse, Z. The nutritional status and digestibility of Oreochromis niloticus L. diet in Lake Langeno, Ethiopia. *Hydrobiologia* **416**, 97-106 (1999).
200. Rennella, A. & Quirós, R. The effects of hydrology on plankton biomass in shallow lakes of the Pampa Plain. *Hydrobiologia* **556**, 181-191 (2006).
201. Woelfl, S. The distribution of large mixotrophic ciliates (< i> Stentor</i>) in deep North Patagonian lakes (Chile): First results. *Limnologica-Ecology and Management of Inland Waters* **37**, 28-36 (2007).
202. Clemente, J.M., Mazzeo, N., Gorga, J. & Meerhoff, M. Succession and collapse of macrozoobenthos in a subtropical hypertrophic lake under restoration (Lake Rodó, Uruguay). *Aquatic ecology* **39**, 455-464 (2005).
203. Liikanen, A., Huttunen, J.T., Murtoniemi, T., Tanskanen, H., Väisänen, T., Silvola, J., Alm, J. & Martikainen, P.J. Spatial and seasonal variation in greenhouse gas and nutrient dynamics and their interactions in the sediments of a boreal eutrophic lake. *Biogeochemistry* **65**, 83-103 (2003).
204. Fytianos, K. & Kotzakioti, A. Sequential fractionation of phosphorus in lake sediments of Northern Greece. *Environmental Monitoring and Assessment* **100**, 191-200 (2005).
205. Husen, M.A. & Dhakal, R.P. Seasonal variations of zooplankton, chlorophyll-a and nutrients in Phewa Lake, Pokhara Valley, Nepal. *Ecoprint: An International Journal of Ecology* **16**, 51-57

(2009).
206. Voigt, R., Grüger, E., Baier, J. & Meischner, D. Seasonal variability of Holocene climate: a palaeolimnological study on varved sediments in Lake Jues (Harz Mountains, Germany). *Journal of Paleolimnology* **40**, 1021-1052 (2008).
207. Torremorell, A., Bustigorry, J., Escaray, R. & Zagarese, H.E. Seasonal dynamics of a large, shallow lake, laguna Chascomús: the role of light limitation and other physical variables. *Limnologica-Ecology and Management of Inland Waters* **37**, 100-108 (2007).
208. Souza, M.B.G., Barros, C.F., Barbosa, F., Hajnal, É. & Padisák, J. Role of atelomixis in replacement of phytoplankton assemblages in Dom Helvécio Lake, South-East Brazil. *Hydrobiologia* **607**, 211-224 (2008).
209. Oberholster, P., Myburgh, J., Ashton, P. & Botha, A.-M. Responses of phytoplankton upon exposure to a mixture of acid mine drainage and high levels of nutrient pollution in Lake Loskop, South Africa. *Ecotoxicology and environmental safety* **73**, 326-335 (2010).
210. Downs, T.M., Schallenberg, M. & Burns, C.W. Responses of lake phytoplankton to micronutrient enrichment: a study in two New Zealand lakes and an analysis of published data. *Aquatic Sciences* **70**, 347-360 (2008).
211. Schallenberg, M. & Burns, C.W. A temperate, tidal lake ‐ wetland complex 2. Water quality and implications for zooplankton community structure. *New Zealand Journal of Marine and Freshwater Research* **37**, 429-447 (2003).
212. Kirschner, A.K., Schlesinger, J., Farnleitner, A.H., Hornek, R., Süß, B., Golda, B., Herzig, A. & Reitner, B. Rapid growth of planktonic Vibrio cholerae non-O1/non-O139 strains in a large alkaline lake in Austria: dependence on temperature and dissolved organic carbon quality. *Applied and environmental microbiology* **74**, 2004-2015 (2008).
213. Skipperud, L., Jørgensen, A., Heier, L., Salbu, B. & Rosseland, B. Po-210 and Pb-210 in water and fish from Taboshar uranium mining Pit Lake, Tajikistan. *Journal of environmental radioactivity* **123**, 82-89 (2013).
214. Modenutti, B. & Pérez, G. Planktonic ciliates from an oligotrophic south Andean lake, Morenito lake (Patagonia, Argentina). *Brazilian Journal of Biology* **61**, 389-395 (2001).
215. Sarmento, H., Isumbisho, M., Stenuite, S., Darchambeau, F., Leporcq, B. & Descy, J.-P. Phytoplankton ecology of Lake Kivu (eastern Africa): biomass, production and elemental ratios. *Verhandlungen des Internationalen Verein Limnologie* **30**, 709-713 (2009).
216. DESCY, J.P., HARDY, M.A., Stenuite, S., Pirlot, S., Leporcq, B., Kimirei, I., Sekadende, B., Mwaitega, S. & Sinyenza, D. Phytoplankton pigments and community composition in Lake Tanganyika. *Freshwater Biology* **50**, 668-684 (2005).
217. Guildford, S.J. & Hecky, R.E. Total nitrogen, total phosphorus, and nutrient limitation in lakes and oceans: Is there a common relationship? *Limnology And Oceanography* **45**, 1213-1223 (2000).
218. Pacheco, J.P., Iglesias, C., Meerhoff, M., Fosalba, C., Goyenola, G., Teixeira-de Mello, F., García, S., Gelós, M. & García-Rodríguez, F. Phytoplankton community structure in five subtropical shallow lakes with different trophic status (Uruguay): a morphology-based approach. *Hydrobiologia* **646**, 187-197 (2010).
219. Pasztaleniec, A. & Poniewozik, M. Phytoplankton based assessment of the ecological status of four shallow lakes (Eastern Poland) according to Water Framework Directive–a comparison of approaches. *Limnologica-Ecology and Management of Inland Waters* **40**, 251-259 (2010).
220. Allende, L., Tell, G., Zagarese, H., Torremorell, A., Pérez, G., Bustingorry, J., Escaray, R. & Izaguirre,

I. Phytoplankton and primary production in clear-vegetated, inorganic-turbid, and algal-turbid shallow lakes from the pampa plain (Argentina). *Hydrobiologia* **624**, 45-60 (2009).

221. Raut, R., Sharma, S., Bajracharya, R.M., Sharma, C.M. & Gurung, S. Physico-chemical Characterization of Gosainkunda Lake. *Nepal Journal of Science and Technology* **13**, 107-114 (2013).
222. Ribeiro, D., Martins, G., Nogueira, R., Cruz, J.V. & Brito, A. Phosphorus fractionation in volcanic lake sediments (Azores–Portugal). *Chemosphere* **70**, 1256-1263 (2008).
223. Kaiserli, A., Voutsa, D. & Samara, C. Phosphorus fractionation in lake sediments–Lakes Volvi and Koronia, N. Greece. *Chemosphere* **46**, 1147-1155 (2002).
224. Rae, R., Howard-Williams, C., Hawes, I., Schwarz, A.-M. & Vincent, W.F. Penetration of solar ultraviolet radiation into New Zealand lakes: influence of dissolved organic carbon and catchment vegetation. *Limnology* **2**, 79-89 (2001).
225. Paul, A. & Peters, R.H. Patterns in planktonic P: R ratios in lakes: Influence of lake trophy and dissolved organic carbon. *Limnol. Oceanogr* **39**, 772-787 (1994).
226. Shinneman, A.L., Almendinger, J.E., Umbanhowar, C.E., Edlund, M.B. & Nergui, S. Paleolimnologic evidence for recent eutrophication in the Valley of the Great Lakes (Mongolia). *Ecosystems* **12**, 944-960 (2009).
227. Pérez, G.L., Torremorell, A., Bustingorry, J., Escaray, R., Pérez, P., Diéguez, M. & Zagarese, H. Optical characteristics of shallow lakes from the Pampa and Patagonia regions of Argentina. *Limnologica-Ecology and Management of Inland Waters* **40**, 30-39 (2010).
228. Haberman, J. & Laugaste, R. On characteristics reflecting the trophic state of large and shallow Estonian lakes (L. Peipsi, L. Võrtsjärv). *Hydrobiologia* **506**, 737-744 (2003).
229. Soto, D. Oligotrophic patterns in southern Chilean lakes: the relevance of nutrients and mixing depth. *Revista Chilena de Historia Natural* **75**, 377-393 (2002).
230. Okbah, M. Nitrogen and phosphorus species of Lake Burullus water (Egypt). (2005).
231. Ogier, S., Disnar, J.-R., Albéric, P. & Bourdier, G. Neutral carbohydrate geochemistry of particulate material (trap and core sediments) in an eutrophic lake (Aydat, France). *Organic Geochemistry* **32**, 151-162 (2001).
232. Salmaso, N. & Padisák, J. Morpho-functional groups and phytoplankton development in two deep lakes (Lake Garda, Italy and Lake Stechlin, Germany). *Hydrobiologia* **578**, 97-112 (2007).
233. Krevs, A., Kucinskiene, A. & Paskauskas, R. Microbial destruction of organic matter in the bottom sediments of Lithuanian lakes. *Microbiology* **75**, 213-218 (2006).
234. Power, M., Klein, G., Guiguer, K. & Kwan, M. Mercury accumulation in the fish community of a sub‐Arctic lake in relation to trophic position and carbon sources. *Journal of Applied Ecology* **39**, 819-830 (2002).
235. LØVIK, J.E. & KJELLBERG, G. Long-term changes of the crustacean zooplankton community in Lake Mjøsa, the largest lake in Norway. *Journal of Limnology* **62**, 143-150 (2003).
236. Hart, R.C. & Boane, C. Limnology of southern African coastal lakes—new vistas from Mozambique. *African Journal of Aquatic Science* **29**, 145-159 (2004).
237. Khondker, M., Alfasane, M.A., Islam, M.S., Bhuiyan, M.A.H. & Gani, M.A. Limnology of Lake Bogakain, Bandarban, Bangladesh. *Bangladesh Journal of Botany* **39**, 153-159 (2010).
238. Gunkel, G. & Casallas, J. Limnology of an equatorial high mountain lake—Lago San Pablo, Ecuador: The significance of deep diurnal mixing for lake productivity. *Limnologica-Ecology and Management of Inland Waters* **32**, 33-43 (2002).

239. Gunkel, G. Limnology of an equatorial high mountain lake in Ecuador, Lago San Pablo. *Limnologica-Ecology and Management of Inland Waters* **30**, 113-120 (2000).
240. Ostapenia, A.P., Parparov, A. & Berman, T. Lability of organic carbon in lakes of different trophic status. *Freshwater biology* **54**, 1312-1323 (2009).
241. KASPEROVICIENE, J., KOREIVIENE, J. & PASKAUSKAS, R. Cyanoprokaryotes and microcystins dynamics in shallow hypertrophic lake (South-Eastern Lithuania). *Oceanological and Hydrobiological Studies* **34**, 93-104 (2005).
242. Simčič, T. & Brancelj, A. Intensity of mineralization processes in mountain lakes in NW Slovenia. *Aquatic Ecology* **36**, 345-354 (2002).
243. Schmidt, R., Kamenik, C., Lange-Bertalot, H. & Rolf, K. Fragilaria and Staurosira (Bacillariophyceae) from sediment surfaces of 40 lakes in the Austrian Alps in relation to environmental variables, and their potential for palaeoclimatology. *Journal of Limnology* **63**, 171-189 (2004).
244. Murase, J. & Sakamoto, M. Horizontal distribution of carbon and nitrogen and their isotopic
245. Grey, J., Kelly, A. & Jones, R.I. High intraspecific variability in carbon and nitrogen stable isotope ratios of lake chironomid larvae. *Limnology and Oceanography* **49**, 239-244 (2004).
246. Mankiewicz, J., Komarkova, J., Izydorczyk, K., Jurczak, T., Tarczynska, M. & Zalewski, M. Hepatotoxic cyanobacterial blooms in the lakes of northern Poland. *Environmental toxicology* **20**, 499-506 (2005).
247. Usanzineza, D., Nhapi, I., Gashagaza, J. & Kashaigili, J. Heavy metal pollution and nutrient levels in Lake Muhazi, Rwanda.
248. Hanson, P.C., Bade, D.L., Carpenter, S.R. & Kratz, T.K. Lake metabolism: Relationships with dissolved organic carbon and phosphorus. *Limnology and Oceanography* **48**, 1112-1119 (2003).
249. Balseiro, E.G., Queimaliños, C.P. & Modenutti, B.E. Grazing impact on autotrophic picoplankton in two south Andean lakes (Patagonia, Argentina) with different light: nutrient ratios. *Rev. Chil. Hist. Nat* **77**, 73-85 (2004).
250. Hwang, S.-J., Kim, H.-S., Shin, J.-K., Oh, J.-M. & Kong, D.-S. Grazing effects of a freshwater bivalve (Corbicula leana Prime) and large zooplankton on phytoplankton communities in two Korean lakes. *Hydrobiologia* **515**, 161-179 (2004).
251. Valdovinos, C. & Pedreros, P. Geographic variations in shell growth rates of the mussel< i> Diplodon chilensis</i> from temperate lakes of Chile: Implications for biodiversity conservation. *Limnologica-Ecology and Management of Inland Waters* **37**, 63-75 (2007).
252. Olin, M., Rask, M., Ruuhljärvi, J., Kurkilahti, M., Ala‐Opas, P. & Ylönen, O. Fish community structure in mesotrophic and eutrophic lakes of southern Finland: the relative abundances of percids and cyprinids along a trophic gradient. *Journal of Fish Biology* **60**, 593-612 (2002).
253. Torres, I.C., Inglett, K.S. & Reddy, K. Heterotrophic microbial activity in lake sediments: effects of organic electron donors. *Biogeochemistry* **104**, 165-181 (2011).
254. Arts, M.T., Robarts, R.D., Kasai, F., Waiser, M.J., Tumber, V.P., Plante, A.J., Rai, H. & de Lange, H.J. The attenuation of ultraviolet radiation in high dissolved organic carbon waters of wetlands and lakes on the northern Great Plains. *Limnology and Oceanography* **45**, 292-299 (2000).
255. Carignan, R., Planas, D. & Vis, C. Planktonic production and respiration in oligotrophic Shield lakes. *Limnology And Oceanography* **45**, 189-199 (2000).
256. Curtis, P.J. & Schindler, D.W. Hydrologic control of dissolved organic matter in low-order Precambrian Shield lakes. *Biogeochemistry* **36**, 125-138 (1997).
257. D'Arcy, P. & Carignan, R. Influence of catchment topography on water chemistry in southeastern

Quebec Shield lakes. *Canadian Journal of Fisheries and Aquatic Sciences* **54**, 2215-2227 (1997).
258. Dillon, P.J. & Molot, L.A. Dissolved organic and inorganic carbon mass balances in central Ontario lakes. *Biogeochemistry* **36**, 29-42 (1997).
259. Del Giorgio, P.A., Cole, J.J., Caraco, N.F. & Peters, R.H. Linking planktonic biomass and metabolism to net gas fluxes in northern temperate lakes. *Ecology* **80**, 1422-1431 (1999).
260. Górniak, A., Jekatierynczuk - Rudczyk, E. & Dobrzyń, P. Hydrochemistry of three dystrophic lakes in northeastern Poland. *Acta hydrochimica et hydrobiologica* **27**, 12-18 (1999).
261. Hope, D., Kratz, T.K. & Riera, J.L. Relationship between pCO2 and dissolved organic carbon in northern Wisconsin lakes. *Journal of environmental quality* **25**, 1442-1445 (1996).
262. Jonsson, A., Karlsson, J. & Jansson, M. Sources of carbon dioxide supersaturation in clearwater and humic lakes in northern Sweden. *Ecosystems* **6**, 224-235 (2003).
263. Kelly, C.A., Fee, E., Ramlal, P.S., Rudd, J.W., Hesslein, R.H., Anema, C. & Schindler, E.U. Natural variability of carbon dioxide and net epilimnetic production in the surface waters of boreal lakes of different sizes. *Limnology and Oceanography* **46**, 1054-1064 (2001).
264. Laurion, I., Ventura, M., Catalan, J., Psenner, R. & Sommaruga, R. Attenuation of ultraviolet radiation in mountain lakes: factors controlling the among-and within-lake variability. *Limnology and Oceanography* **45**, 1274-1288 (2000).
265. Lim, D.S., Douglas, M.S., Smol, J.P. & Lean, D.R. Physical and chemical limnological characteristics of 38 lakes and ponds on Bathurst Island, Nunavut, Canadian High Arctic. *International Review of Hydrobiology* **86**, 1-22 (2001).
266. Michelutti, N., Douglas, M.S., Muir, D.C., Wang, X. & Smol, J.P. Limnological Characteristics of 38 Lakes and Pondson Axel Heiberg Island, High Arctic Canada. *International Review of Hydrobiology* **87**, 385-399 (2002).
267. Pienitz, R., Smol, J.P. & Lean, D.R. Physical and chemical limnology of 24 lakes located between Yellowknife and Contwoyto Lake, Northwest Territories (Canada). *Canadian Journal of Fisheries and Aquatic Sciences* **54**, 347-358 (1997).
268. Pienitz, R., Smol, J.P. & Lean, D.R. Physical and chemical limnology of 59 lakes located between the southern Yukon and the Tuktoyaktuk Peninsula, Northwest Territories (Canada). *Canadian Journal of Fisheries and Aquatic Sciences* **54**, 330-346 (1997).
269. Sabbe, K., Hodgson, D.A., Verleyen, E., Taton, A., Wilmotte, A., Vanhoutte, K. & Vyverman, W. Salinity, depth and the structure and composition of microbial mats in continental Antarctic lakes. *Freshwater Biology* **49**, 296-319 (2004).
270. Scully, N., McQueen, D., Lean, D. & Cooper, W. Hydrogen peroxide formation: The interaction of ultraviolet radiation and dissolved organic carbon in lake waters along a 43-75 N gradient. *Limnology and Oceanography* **41**, 540-548 (1996).
271. Pacheco, F.S., Roland, F. & Downing, J.A. Eutrophication reverses whole-lake carbon budgets. *Inland Waters* **4**, 41-48 (2013).
272. Gudasz, C., Bastviken, D., Premke, K., Steger, K. & Tranvik, L.J. Constrained microbial processing of allochthonous organic carbon in boreal lake sediments. *Limnology And Oceanography* **57**, 163-175 (2012).
273. Stets, E.G., Striegl, R.G. & Aiken, G.R. Dissolved organic carbon export and internal cycling in small, headwater lakes. *Global Biogeochemical Cycles* **24** (2010).
274. Nixdorf, B. & Deneke, R. Why'very shallow 'lakes are more successful opposing reduced nutrient loads. *Hydrobiologia* **342**, 269-284 (1997).

275. The Eastern Lake Survey - Phase I (ELS-I). Surface waters data and metadata files. *U.S. Environmental Protection Agency*, Available online at http://www.epa.gov/emfjulte/html/data/surfwatr/data/els.html. (1984).
276. The Western Lake Survey-Phase I (WLS-I). Surface waters data and metadata files. *U.S. Environmental Protection Agency*, Available from http://www.epa.gov/emfjulte/html/data/surfwatr/data/wls.html. (1985).
277. Swedish national lake monitoring. Riksinventering 2000, vattenkemi i sjöar. Institutionen för miljöanalys. Available from http://info1.ma.slu.se/RI2000/. (2000).
278. European environment agency. Waterbase—lakes Version 14. Available from http://www.eea.europa.eu/data-and-maps/data/waterbase-lakes-10#tab-european-data.
279. Finlay, J.C., Small, G.E. & Sterner, R.W. Human Influences on Nitrogen Removal in Lakes. *Science* **342**, 247-250 (2013).

Table S1. Summary of data for countries/regions.

Country/Region	No. of analyzed lakes
Albania	4
Antarctica	28
Arctic Canada	57
Argentina	147
Austria	91
Bangladesh	1
Belarus	3
Belgium	5
Bosnia and Herzegovina	10
BRAZIL	5
Bulgaria	30
Canada	362
Chile	60
china	53
Croatia	9
Cyprus	9
Democratic Republic of Congo	1
Denmark	67
Ecuador	1
Egypt	1
Estonia	19
Ethiopia	1
Finland	273
France	137
Germany	79
Greece	16
Hungary	24
Iceland	39
India	1
Ireland	149
Israel	1
Italy	261
Japan	4
Korea	2
Kosovo under the UNSCR 1244/99	4
Latvia	74
Lithuania	51
Macedonia, the Former Yugoslav Republic of	2
Malta	2
Mongolia	5
Montenegro	3
Mozambique	14

Nepal	2
Netherlands	15
New Zealand	21
Norway	162
POLAND	345
Portugal	35
Romania	17
Russia	1
Rwanda	1
Senegal	1
Serbia	29
Slovakia	34
Slovenia	32
South Africa	8
Spain	470
Sweden	2770
Switzerland	55
Tajikistan	1
Tanzania	1
The Republic of Malawi	1
Turkey	3
Ugandan	6
United Kingdom	157
Uruguay	21
USA	2052
Zimbabwe	1

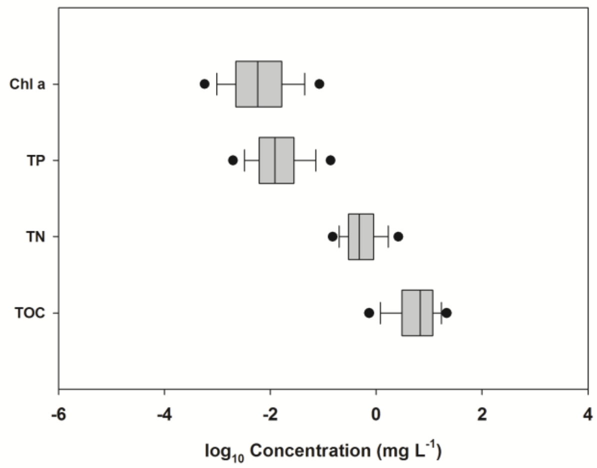

Fig. S1. Box- and whisker plot showing TOC, TN, TP and Chl *a* concentrations in global lake water.

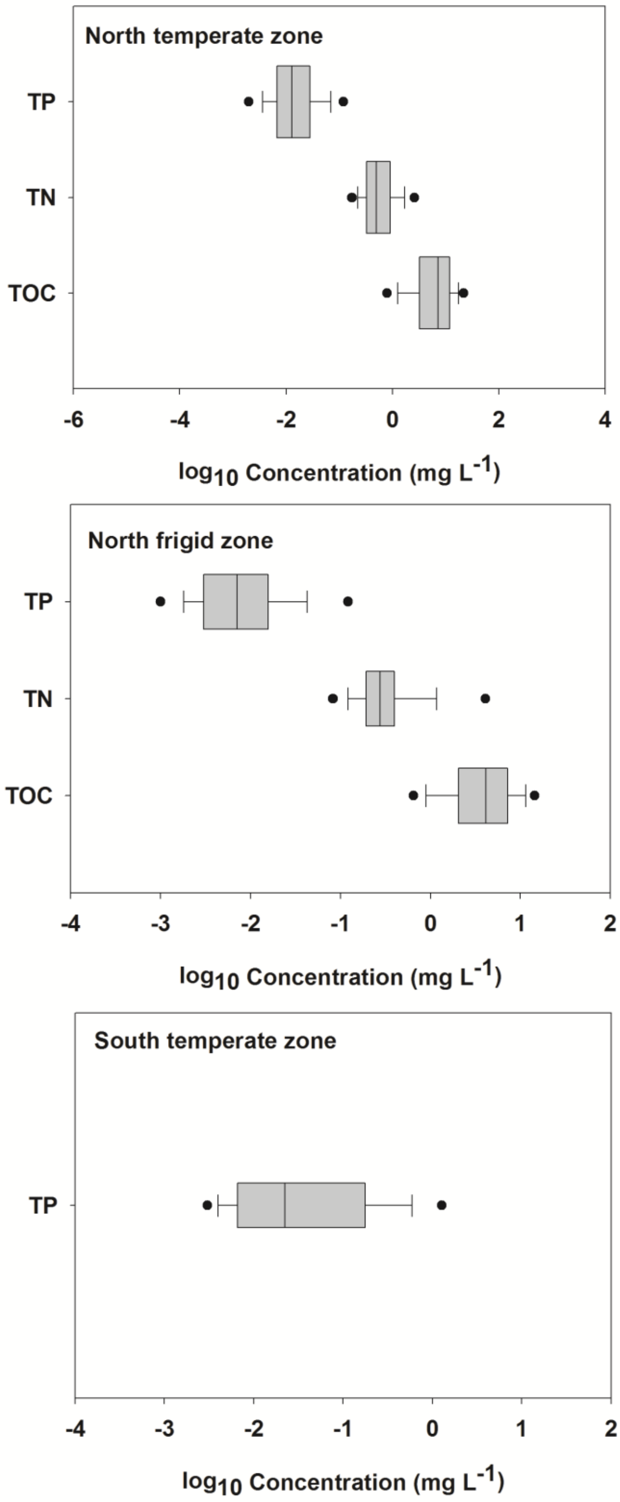

Fig. S2. Box- and whisker plot showing TOC, TN and TP concentrations for lakes divided into different climate zones.

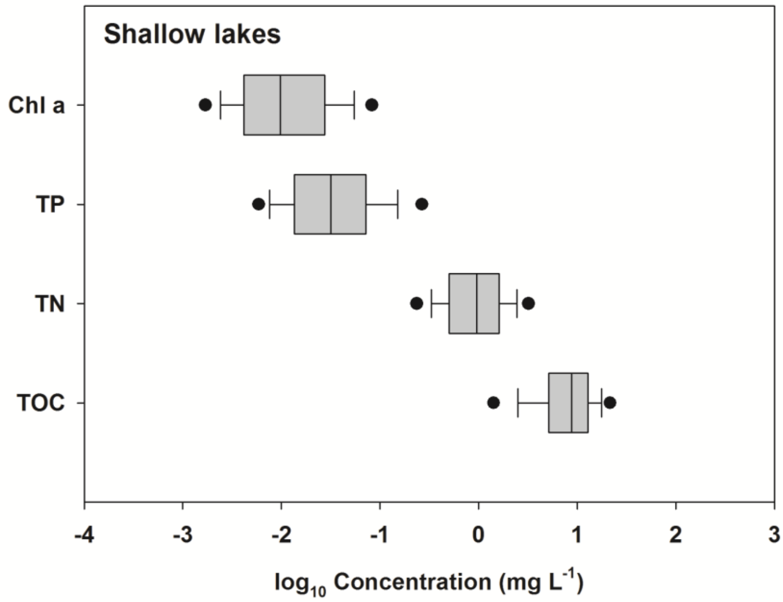

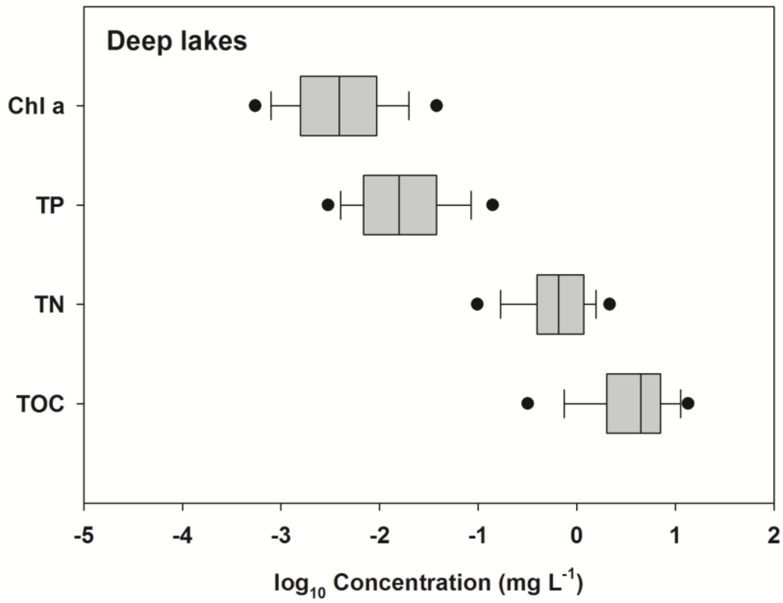

Fig. S3. Box- and whisker plot showing TOC, TN, TP and Chl *a* concentrations in shallow and deep lakes.

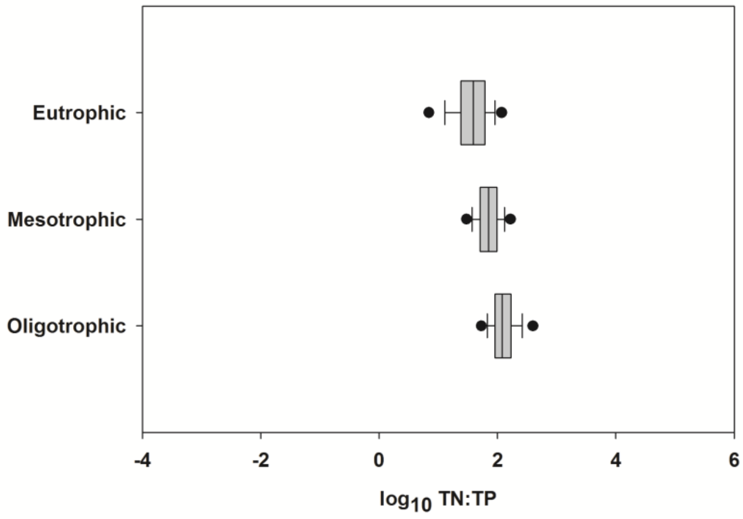

Fig. S4. Box- and whisker plot showing TN:TP ratios in lakes with different trophic states.

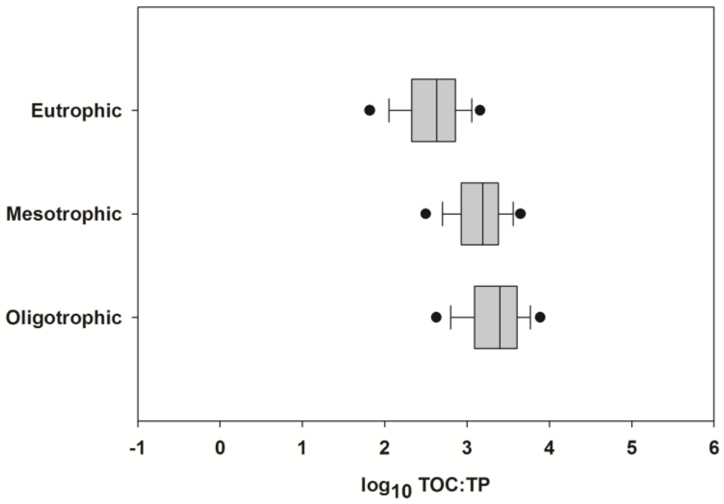

Fig. S5. Box- and whisker plot showing TOC:TP ratios in lakes with different trophic states.

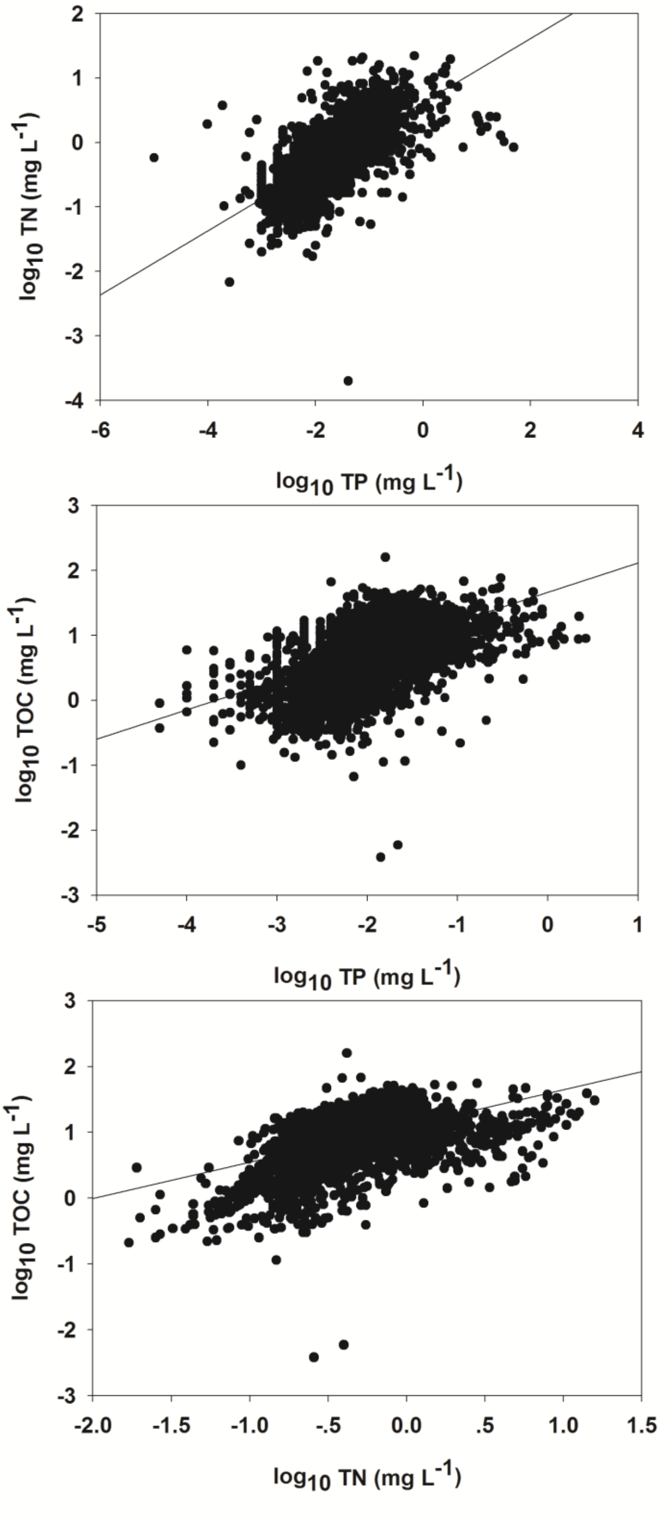

Fig. S6. Relationship between TOC, TN and TP concentrations in global lake water ($\log_{10}$ TN = 0.619 + 0.497$\log_{10}$ TP, R^2 = 0.513, P < 0.001; $\log_{10}$ TOC = 1.663 + 0.452$\log_{10}$ TP, R^2 = 0.249, P < 0.001; $\log_{10}$ TOC = 1.097 + 0.550$\log_{10}$ TN, R^2 = 0.239, P < 0.001).

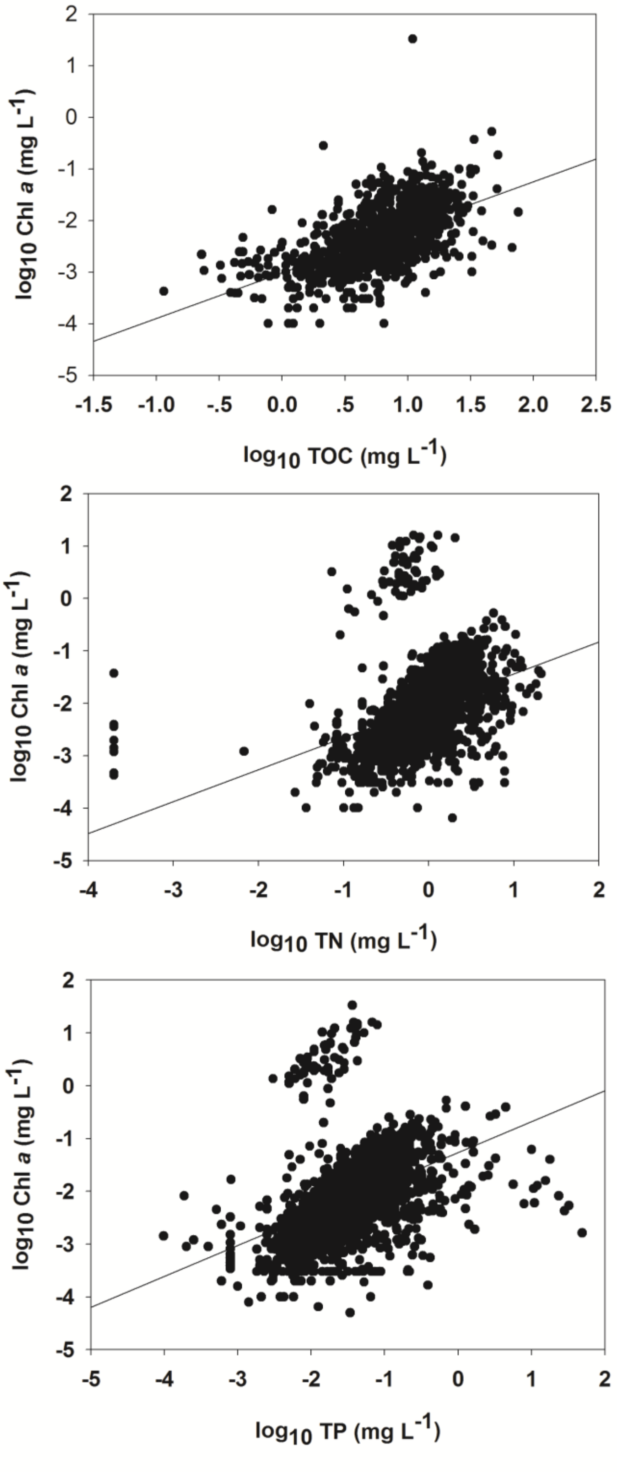

Fig. S7. Influence of TOC, TN and TP concentrations on Chl *a* in global lake water ($\log_{10}$ Chl $a = -3.014 + 0.884\log_{10}$ TOC, $R^2 = 0.322$, $P < 0.001$; $\log_{10}$ Chl $a = -2.046 + 0.608\log_{10}$ TN, $R^2 = 0.155$, $P < 0.001$; $\log_{10}$ Chl $a = -1.268 + 0.586\log_{10}$ TP, $R^2 = 0.220$, $P < 0.001$).

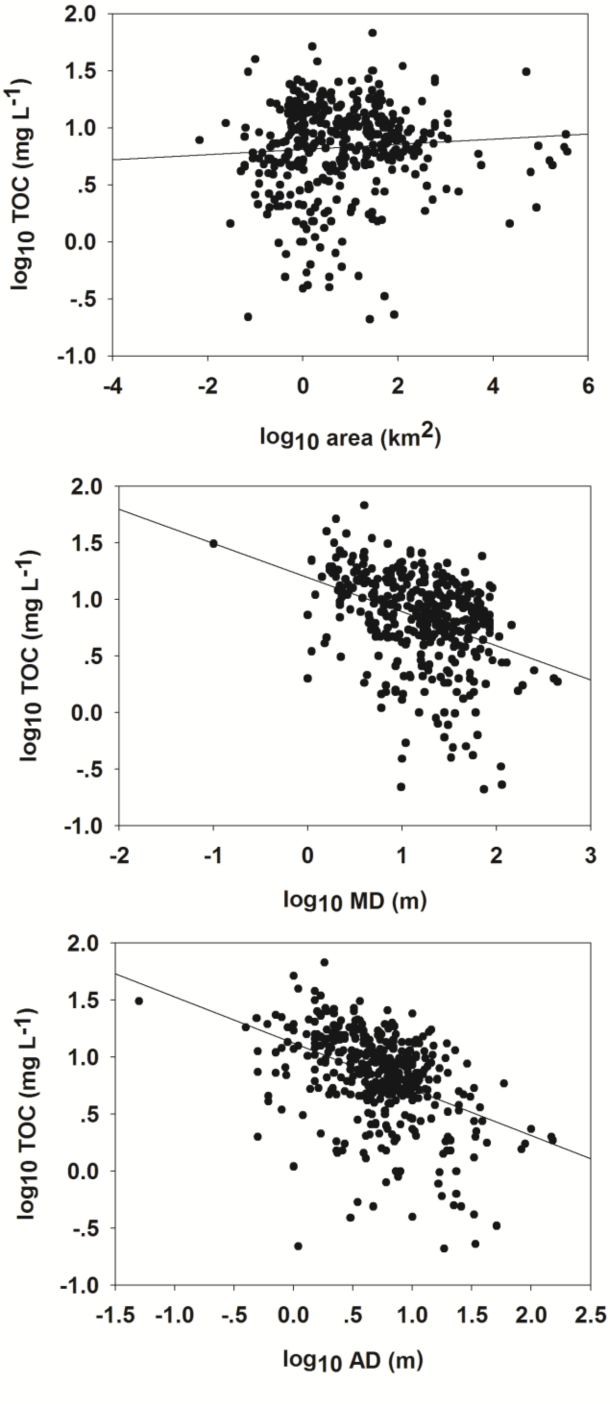

Fig. S8. Influence of morphological characteristics on TOC concentrations in global lake water ($\log_{10}$ TOC = 0.811 + 0.022$\log_{10}$ area, R^2 = 0.005, P > 0.05; $\log_{10}$ TOC = 1.192 - 0.302$\log_{10}$ MD, R^2 = 0.147, P < 0.001; $\log_{10}$ TOC = 1.122 - 0.405$\log_{10}$ AD, R^2 = 0.188, P < 0.001; MD, maximum depth; AD, average depth).

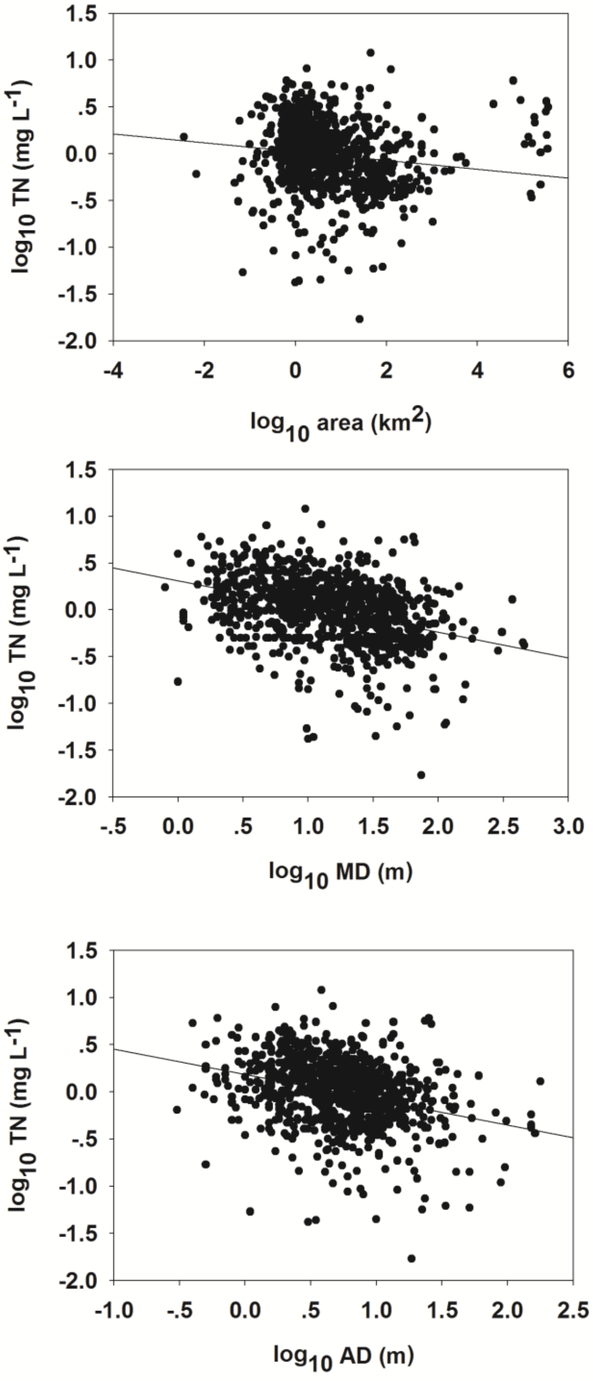

Fig. S9. Influence of morphological characteristics on TN concentrations in global lake water ($\log_{10}$ TN = 0.022 - 0.047$\log_{10}$ area, R^2 = 0.020, P < 0.001; $\log_{10}$ TN = 0.313 - 0.275$\log_{10}$ MD, R^2 = 0.141, P < 0.001; $\log_{10}$ TN = 0.185 - 0.268$\log_{10}$ AD, R^2 = 0.098, P < 0.001; MD, maximum depth; AD, average depth).

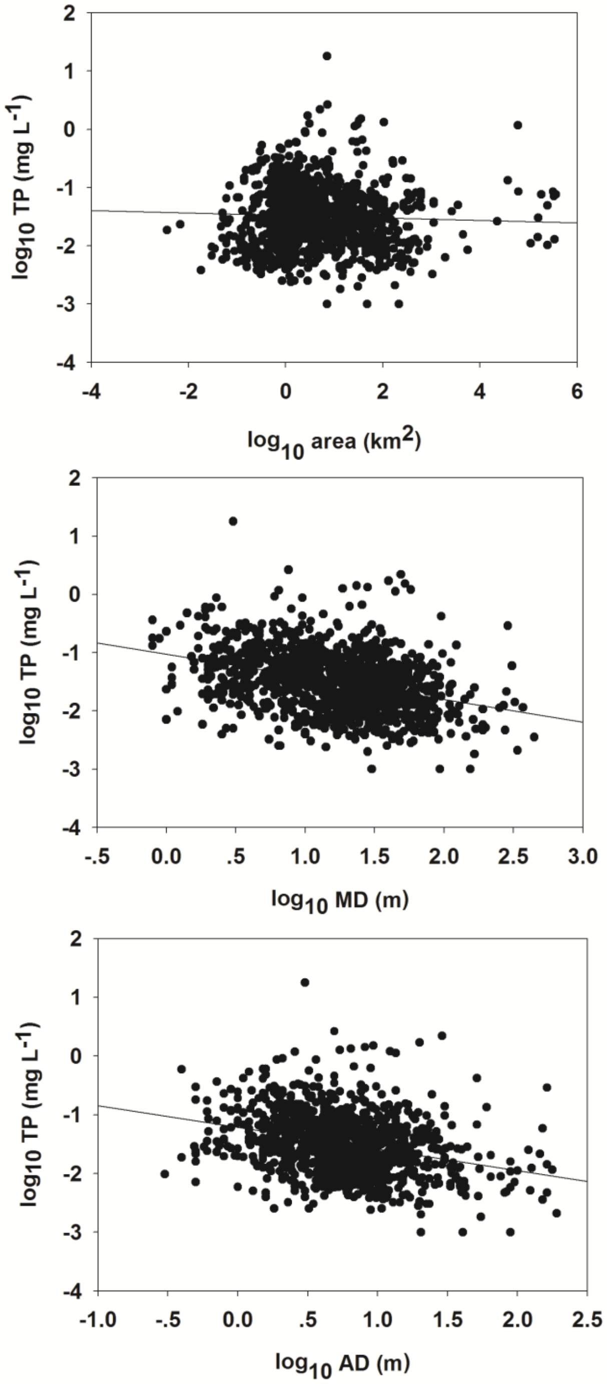

Fig. S10. Influence of morphological characteristics on TP concentrations in global lake water ($\log_{10}$ TP = -1.478 - 0.021$\log_{10}$ area, R^2 = 0.002, P > 0.05; $\log_{10}$ TP = -1.027 - 0.389$\log_{10}$ MD, R^2 = 0.130, P < 0.001; $\log_{10}$ TP = -1.213 - 0.370$\log_{10}$ AD, R^2 = 0.094, P < 0.001; MD, maximum depth; AD, average depth).

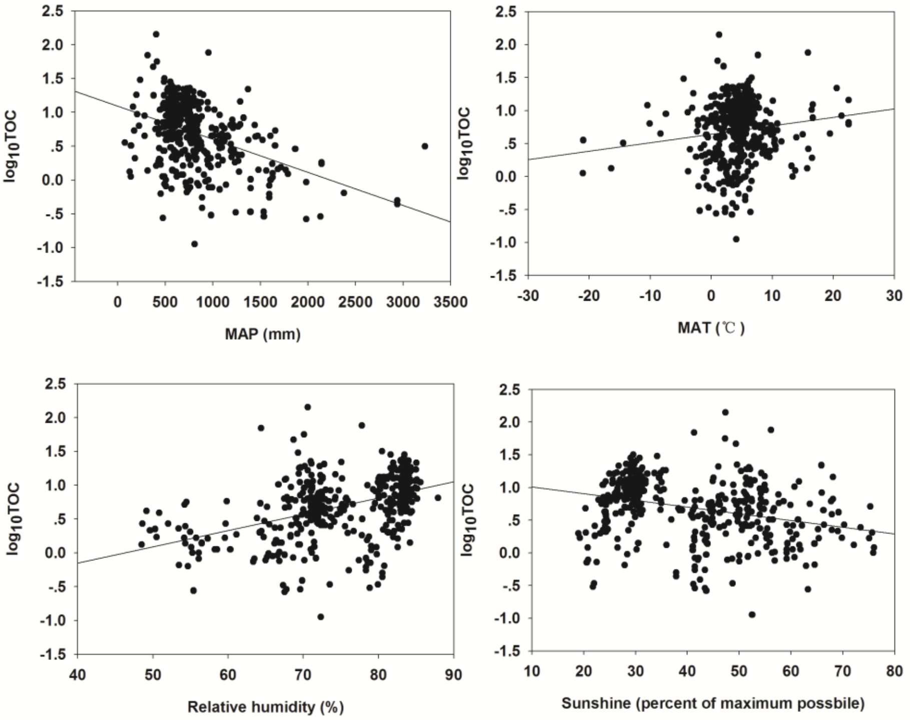

Fig. S11. Influence of climate on TOC concentrations in global lake water ($\log_{10}$ TOC = 1.092 - 0.0005MAP, R^2 = 0.176, P < 0.001; $\log_{10}$ TOC = 0.640 + 0.013MAT, R^2 = 0.017, P < 0.01; $\log_{10}$ TOC = -1.115 + 0.024RH, R^2 = 0.196, P < 0.001; $\log_{10}$ TOC = 1.113 - 0.010SUN, R^2 = 0.088, P < 0.001; MAT, temperature, MAP, precipitation; SUN, sunshine; RH, relative humidity).

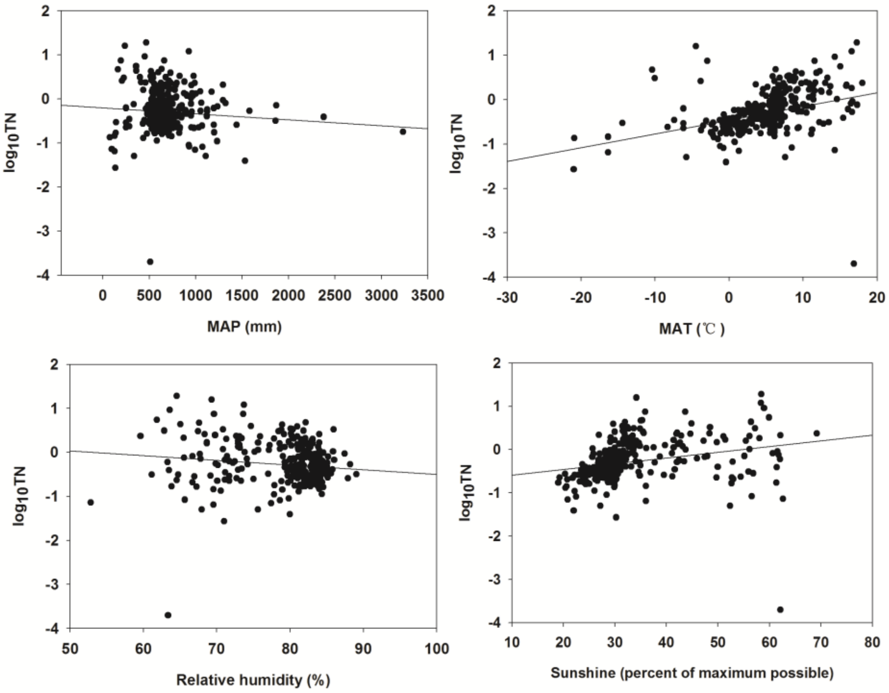

Fig. S12. Influence of climate on TN concentrations in global lake water ($\log_{10}$ TN = -0.204 - 0.0001MAP, R^2 = 0.007, P > 0.05; $\log_{10}$ TN = -0.463 + 0.031MAT, R^2 = 0.126, P < 0.001; $\log_{10}$ TN = 0.570 - 0.011RH, R^2 = 0.021, P < 0.01; $\log_{10}$ TN = -0.725 + 0.013SUN, R^2 = 0.072, P < 0.001; MAT, temperature, MAP, precipitation; SUN, sunshine; RH, relative humidity).

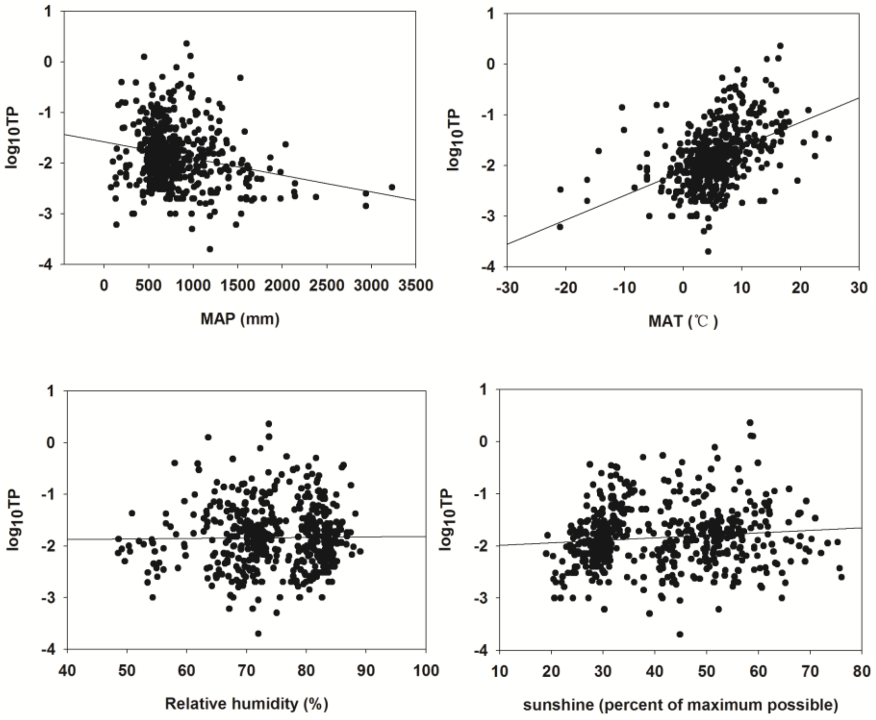

Fig. S13. Influence of climate on TP concentrations in global lake water (log_{10} TP = -1.578 - 0.0003MAP, R^2 = 0.049, P < 0.001; log_{10} TP = -2.109 + 0.048MAT, R^2 = 0.199, P < 0.001; log_{10} TP = -1.915 + 0.001RH, R^2 = 0.000, P =0.741; log_{10} TP = -2.040 + 0.005SUN, R^2 = 0.012, P < 0.05; MAT, temperature, MAP, precipitation; SUN, sunshine; RH, relative humidity).
